# Hmga2 protein loss alters nuclear envelope and 3D chromatin structure

**DOI:** 10.1186/s12915-022-01375-3

**Published:** 2022-08-02

**Authors:** Giuseppina Divisato, Andrea M. Chiariello, Andrea Esposito, Pietro Zoppoli, Federico Zambelli, Maria Antonietta Elia, Graziano Pesole, Danny Incarnato, Fabiana Passaro, Silvia Piscitelli, Salvatore Oliviero, Mario Nicodemi, Silvia Parisi, Tommaso Russo

**Affiliations:** 1 Dipartimento di Medicina molecolare e biotecnologie mediche, Università di Napoli Federico II, Naples, Italy; 2 Dipartimento di Fisica, Università di Napoli Federico II, and INFN Napoli, Naples, Italy; 3 Dipartimento di Bioscienze, Università di Milano Statale, Milan, Italy; 4 Dipartimento Di Bioscienze, Biotecnologie e Biofarmaceutica, Università di Bari A. Moro and IBIOM CNR, Bari, Italy; 5 University of Groningen, GBB Institute, Groningen, The Netherlands; 6 Dipartimento di Scienze della Vita e Biologia dei Sistemi, Università di Torino and IIGM Candiolo, Turin, Italy; 7 Berlin Institute for Medical Systems Biology, Max-Delbrück Centre for Molecular Medicine, Berlin, Germany; 8 CNR-SPIN, Naples, Italy

**Keywords:** High mobility group proteins, Pluripotent stem cells, Topologically associated domains, Lamin, Nuclear envelope, Histone modifications

## Abstract

**Background:**

The high-mobility group Hmga family of proteins are non-histone chromatin-interacting proteins which have been associated with a number of nuclear functions, including heterochromatin formation, replication, recombination, DNA repair, transcription, and formation of enhanceosomes. Due to its role based on dynamic interaction with chromatin, Hmga2 has a pathogenic role in diverse tumors and has been mainly studied in a cancer context; however, whether Hmga2 has similar physiological functions in normal cells remains less explored. Hmga2 was additionally shown to be required during the exit of embryonic stem cells (ESCs) from the ground state of pluripotency, to allow their transition into epiblast-like cells (EpiLCs), and here, we use that system to gain further understanding of normal Hmga2 function.

**Results:**

We demonstrated that Hmga2 KO pluripotent stem cells fail to develop into EpiLCs. By using this experimental system, we studied the chromatin changes that take place upon the induction of EpiLCs and we observed that the loss of Hmga2 affects the histone mark H3K27me3, whose levels are higher in Hmga2 KO cells. Accordingly, a sustained expression of polycomb repressive complex 2 (PRC2), responsible for H3K27me3 deposition, was observed in KO cells. However, gene expression differences between differentiating wt vs Hmga2 KO cells did not show any significant enrichments of PRC2 targets. Similarly, endogenous Hmga2 association to chromatin in epiblast stem cells did not show any clear relationships with gene expression modification observed in Hmga2 KO. Hmga2 ChIP-seq confirmed that this protein preferentially binds to the chromatin regions associated with nuclear lamina. Starting from this observation, we demonstrated that nuclear lamina underwent severe alterations when Hmga2 KO or KD cells were induced to exit from the naïve state and this phenomenon is accompanied by a mislocalization of the heterochromatin mark H3K9me3 within the nucleus. As nuclear lamina (NL) is involved in the organization of 3D chromatin structure, we explored the possible effects of Hmga2 loss on this phenomenon. The analysis of Hi-C data in wt and Hmga2 KO cells allowed us to observe that inter-TAD (topologically associated domains) interactions in Hmga2 KO cells are different from those observed in wt cells. These differences clearly show a peculiar compartmentalization of inter-TAD interactions in chromatin regions associated or not to nuclear lamina.

**Conclusions:**

Overall, our results indicate that Hmga2 interacts with heterochromatic lamin-associated domains, and highlight a role for Hmga2 in the crosstalk between chromatin and nuclear lamina, affecting the establishment of inter-TAD interactions.

**Supplementary Information:**

The online version contains supplementary material available at 10.1186/s12915-022-01375-3.

## Background

Hmga are non-histone chromatin proteins belonging to the high mobility group protein family [1]. Hmga proteins contain three different DNA binding domains, named AT-hooks domains, by which these proteins bind short AT-rich regions on DNA, and an acidic C-terminal tail [2]. The relevance of Hmga2 functions is demonstrated by the phenotypes of mouse loss of function mutants. Indeed, *Hmga2* gene KO causes the “pigmy” phenotype, mainly consisting in reduced body weight, about 40% of the normal weight [3]. This severe phenotype is due to the reduction of the adipose tissue mass and of that of skeletal muscles [4]. Hmga1, a protein similar to Hmga2, probably partially compensates for the absence of Hmga2: indeed, the *Hmga1/Hmga2* double KO mice have a dramatic phenotype with a 70% reduction of the body weight at birth, not compatible with pups survival [5]. In addition, *Hmga1/Hmga2* double KO also causes a certain degree of embryonic lethality, thus confirming the crucial role of these proteins during development [5].

Hmga proteins appear to dynamically interact with chromatin [6] and a vast literature indicates that they could be involved in various nuclear functions, among these heterochromatin formation, replication, recombination, and DNA repair [7–9]. Hmga proteins compete with histone H1 for the binding to internucleosomal linker DNA [10] and they also influence transcription, contributing to the assembly of complexes at initiator sequences [11] and to the formation of enhanceosomes [12–14].

Hmga2 is expressed during embryogenesis; its levels are undetectable in most adult cells, while it is strongly upregulated in cancer tissues [15]. Many studies aimed at addressing the functions of Hmga2 have been done in cancer cells, mostly because of its possible pathogenic role in various types of tumors. Hmga2 has a physiologic role in ESCs where it is induced upon differentiation and whose suppression alters the exit of ESCs from the ground state of pluripotency, preventing their transition into the primed state of pluripotency, i.e., into EpiLCs [16]. Thus, the transition from ESCs into EpiLCs represent an ideal experimental system where to study the functions of Hmga2 in a normal environment.

In this study, we observed that, in epiblast stem cells (EpiSCs), endogenous Hmga2 is mostly associated with heterochromatin regions interacting with Lamins and we demonstrated that, in pluripotent stem cells (PSCs) induced to exit from the naïve state, the loss of Hmga2 impacts the deposition and distribution of histone modifications and the integrity of the nuclear envelope. These results led to demonstrate that Hmga2 is crucial to guarantee the proper 3D structure of the chromatin, maintaining the right interactions among TADs inside the nucleus and their relationship with Lamin-associated chromatin domains.

## Results

### The absence of Hmga2 alters H3K27me3 deposition upon the induction of the transition from undifferentiated pluripotent stem cells into EpiLCs

**Fig. 1:**
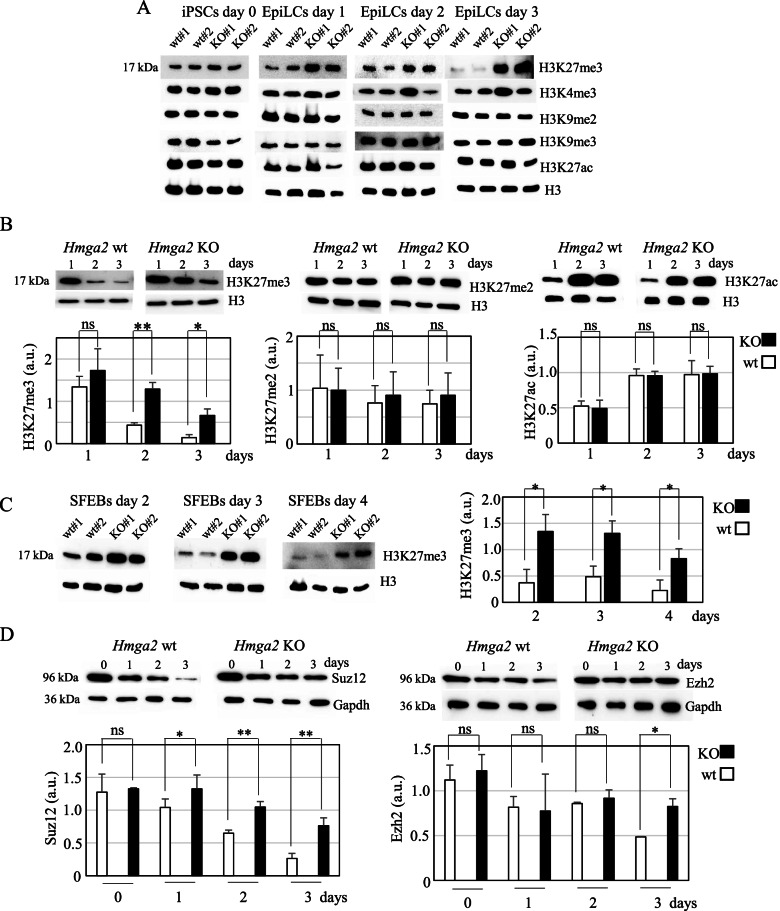
Perturbation of H3K27me3 levels in *Hmga2* wt and KO cells upon the induction of EpiLCs. **A** Western blotting images showing the levels of histone modifications (H3K27me3, H3K4me3, H3K9me2, H3K9me3, and H3K27ac) in wt and KO PSCs at day 0 (undifferentiated cells) and at three different times after the induction of the transition into EpiLCs (day 1, day 2, and day 3). The levels of the histone H3 were used as control. Two wt and two KO clones were used. Quantification and statistical analysis are reported in Additional file 1: Fig. S1B. **B** Western blotting and relative quantification graphs showing the levels of H3K27me3, H3K27me2, and H3K27ac during EpiLC establishment (time course experiments: days 1, 2, and 3) of wt and KO cells. **C** Western blotting images and relative quantification graph showing the levels of H3K27me3 in wt and KO PSCs during SFEB differentiation. **D** Western blotting images and relative quantification graph showing the levels of the PRC2 proteins Suz12 and Ezh2 during EpiLC establishment (time course experiments: days 1, 2, and 3) of wt and KO cells. The data in the graphs represent the mean ± SD (*n*=3 biological replicates) of H3K27me3, H3K27me2, and H3K27ac against total H3 (panels B and C) or of Suz12 and Ezh2 signal intensity against Gapdh expressed as arbitrary units (a.u.). Student’s *t*-test, two tailed (ns: not significant, *p<0.05, **p<0.01)

Considering the general thought that Hmga2 is a chromatin protein, we explored the effects of *Hmga2* suppression (Additional file 1: Fig. S1A) on histone modification dynamics taking place upon the transition of PSCs into EpiLCs. As shown in Fig. 1A (and Additional file 1: Fig. S1B), the trimethylation of K27 of histone H3 (H3K27me3) is affected in *Hmga2* KO cells [16] induced to develop into EpiLCs.

Indeed, while in basal conditions (undifferentiated cells) the levels of H3K27me3 were comparable in Hmga2 wt and KO cells, they were maintained higher in Hmga2 KO cells throughout the three days of EpiLC induction. This difference between wt and KO clones is clearly appreciable in time course experiments, where the expected decrease of H3K27me3 is less pronounced in Hmga2 KO cells than in the wt cells, while the amount of H3K27me2 and H3K27ac showed similar dynamics in both cells (Fig. 1B).

To understand whether the observed effect on the variations of the epigenetic state due to Hmga2 absence is a general mechanism to allow pluripotent stem cells to leave the naïve state, we induced the differentiation of wt and KO Hmga2 PSC clones through the formation serum-free embryoid bodies (SFEBs). This 3D-model mimics different aspects of cell differentiation during early mammalian embryogenesis and it is particularly useful for the interpretation of KO phenotypes, allowing to by-pass the limitations related to 2D-systems [17, 18]. In this experimental setting, the different behavior of H3K27me3 between wt and KO cells was clearly confirmed, with a sustained presence of this histone mark at 2, 3, and 4 days after the induction of differentiation (Fig. 1C). Considering that H3K27me3 modification is catalyzed by polycomb repressive complex 2 (PRC2), we analyzed the expression of two subunits of the core PRC2, Suz12, and Ezh2, and we observed a slight but significant change of Suz12 levels that remained higher in KO cells throughout the induction of the transition (Fig. 1D). A similar behavior, although less pronounced, was also observed for Ezh2 (Fig. 1D).

### Gene expression changes upon the induction of EpiLCs in the absence of Hmga2

**Fig. 2:**
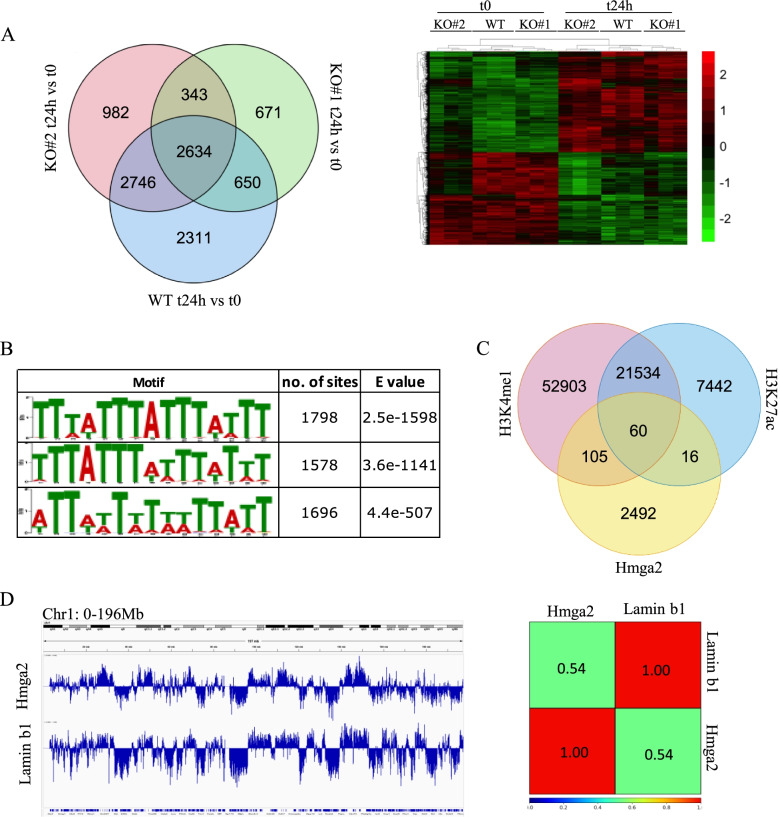
Hmga2 association with chromatin. **A** Comparison of the expression profiles of wt and Hmga2 KO cells induced to differentiate into EpiLCs. The profiles of the two cell types are similar (see also Additional file 2: Table S1) with some differences indicated in the Venn diagram. The heatmap shows the 2634 genes with a common behavior in the three cell clones, according to the Venn diagram. **B** DNA sequence motifs in the regions of the peaks of Chip-seq experiment with chromatin from wt EpiSC, immunoprecipitated with the anti-Hmga2 antibody and analyzed by MEME-chip software with default options. The three consensus sequences emerging from the analysis are shown. **C** Venn diagram of the alignment between Hmga2 peaks and those of chromatin markers H3K4me1 (GSM1382217 from ref. [19]) and H3K27ac (GSM1382219 from ref. [19]). **D** ChIP-seq data of Hmga2 in wt EpiSCs were aligned with available data of DamID in mouse ESCs (ref. [20], GSE17051). The results show a significantly overlap with the regions associated with Lamin b1 (about 51% Spearman correlation). The image shows a 196 Mb region of chromosome 1

The above-described results revealed that the absence of *Hmga2* influences the abundance of the repressive histone modification H3K27me3 upon the exit of PSCs from the undifferentiated naïve state. To address whether this phenomenon is accompanied by a Hmga2-dependent change in the gene expression profile, we performed an RNA sequencing (RNA-seq) experiment to compare *Hmga2* wt and KO cells at 24h after the induction of EpiLCs. The expression profiles of wt cells and two independent clones of *Hmga2* KO cells were similar, with some exceptions (Fig. 2A). Indeed, we found 8341 genes whose expression was significantly modified 24 h after the exit from the naïve state of wt cells. Most of these genes were similarly modified in *Hmga2* KO cells (Additional file 2: Table S1). There were 2311 genes whose expression was significantly modified only in wt cells and in none of the two *Hmga2* KO clones, while the expression of 343 genes was significantly modified only in both *Hmga2* KO clones and not in the wt cells. Then, we compared the list of genes, whose expression changes upon EpiLC induction appeared to be Hmga2-dependent, with the list of PRC2 putative target genes, available in GEO GSE74330 [21]. This comparison showed no significant enrichments.

### Hmga2 association with chromatin in EpiSCs

There are numerous results indicating that Hmga2 interacts with chromatin at specific sites, thus contributing to gene regulation [11–14]. A genome-wide analysis of Hmga2 interaction with chromatin was already done in undifferentiated mouse ESCs overexpressing the recombinant protein [22] that demonstrated a diffuse interaction of Hmga2 with chromatin. We sought to explore endogenous Hmga2 association with chromatin and this was possible in EpiSCs (see the “Methods” section) where the levels of Hmga2 are enough high.

Thus, we performed a ChIP-seq experiment using a primary antibody against Hmga2 and this experiment allowed us to detect a diffuse interaction of endogenous Hmga2 with chromatin, confirming the previously published results obtained in undifferentiated ESCs [22]. In addition, we observed 2673 sites where Hmga2 accumulated as discrete peaks in our non-replicated experiment (2-fold, *p*< 0.001, see Additional file 3: Table S2). The analysis of the sequences of these Hmga2 peaks, by MEME-ChIP software, allowed us to identify three significant motifs (Fig. 2B).

These peaks were preferentially located at the inter-nucleosomal spacers, as demonstrated by the alignment of Hmga2 ChIP-seq with the data deriving from the MNase-seq experiments (ref. [23]) indicating that the number of the overlapping peaks is negligible.

A small number of Hmga2 peaks overlapped or were in close spatial relationship with putative enhancer elements marked by H3K4me1, H3K27ac, or both (Fig. 2C), while no significant overlapping between Hmga2 peaks and H3K27me3 peaks was observed. In all the cases analyzed, the histone marks of the putative enhancers were not significantly modified upon the silencing of *Hmga2* in EpiSCs (Additional file 4: Fig. S2). Furthermore, no significant associations were found between the genes whose transcription start site (TTS) is located within 100 kb from these putative enhancers and the genes whose expression was different in wt vs *Hmga2* KO cells.

The above-reported results do not support a direct relationship between Hmga2 association with chromatin, the observed changes in histone mark deposition and/or with gene expression modification in *Hmga2* KO cells. However, it should be considered that the ChIP-seq experiment was done with chromatin from EpiSCs; thus, a different interaction pattern of Hmga2 during the early steps of this transition cannot be excluded.

### Hmga2 loss alters the structure of the nuclear lamina in differentiating cells

**Fig. 3:**
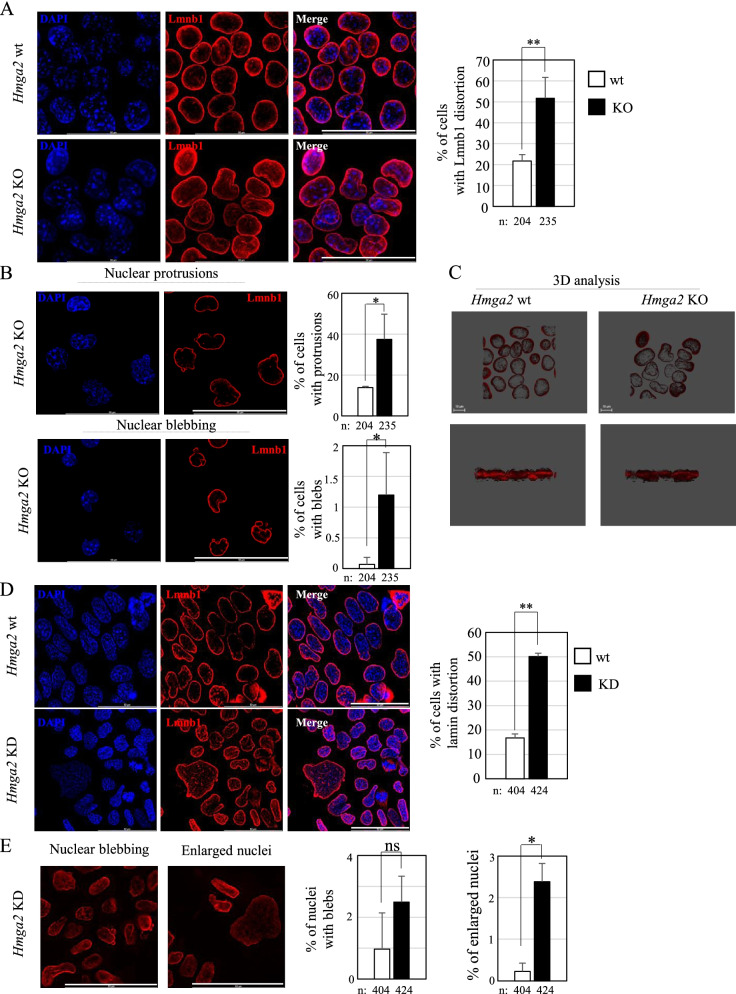
*Hmga2* suppression causes a remarkable distortion of the nuclear lamina. **A** Lmnb1 immunofluorescence (red) on *Hmga2* wt and KO cells at day 1 of EpiLC induction, showing the distortion of the NL in *Hmga2* KO cells. Maximum projection of z-slices (ROI 1024×1024) is shown; quantification graphs obtained by counting >200 nuclei/condition. **B** Representative images (single Z-plane, ROI 1024×1024) of the nuclear abnormalities (nuclear protrusions and nuclear blebbing) observed in *Hmga2* KO cells at day 1 of EpiLC induction and absent in wt cells; quantification graphs obtained by counting >200 nuclei/condition. **C** Three-dimensional analysis of z-slices maximum projections showing the distortion of Lmnb1 (red) as well as the reduced porosity (grey dots inside the NL) detected in *Hmga2* KO cells compared to the wild-type ones. Scale bar= 10 μm. **D** Lmnb1 immunofluorescence (red) on *Hmga2* KD ESCs induced into EpiLCs (day 3) showing an evident distortion of the NL, with some enlarged and wrinkly nuclei. Non-silencing siRNA was used as control. Quantification graphs obtained by counting >400 nuclei/condition. **E** Lmnb1 immunofluorescence on *Hmga2* KD cells showing the nuclear abnormalities (nuclear blebbing, enlarged nuclei) typical of cells devoid of *Hmga2. D*API (blue) was used to counterstain the nuclei. A single plane of the z-stack projection is shown. Scale bars of **A**, **B**, **D**, and **E** = 50 μm. Error bars represent standard deviation. Statistical significance on three biological replicate experiments was determined using Student’s *t*-test (ns: not significant, *p<0.05, **p<0.01)

The analysis of Hmga2 ChIP-seq data showed a significant association (~51% Spearman correlation) of this protein with non-transcribed regions and with Lamin-associated domains (LADs) [24] (Fig. 2D), confirming the results already observed in ESCs overexpressing *Hmga2* [22]. Lamins guarantee the proper structure of the nuclear periphery and are essential to maintain an adequate 3D genome organization [25]. We asked whether the cells lacking Hmga2 suffer from alterations of the NL. Therefore, we performed Lamin b1 (Lmnb1) immunofluorescence on *Hmga2* wild-type and knock-out cells during the transition into EpiLCs. At day 1, *Hmga2* KO cells showed a remarkable distortion of the NL compared to the wt cells, with many nuclear protrusions and a clear nuclear blebbing (Fig. 3A, B).

Tridimensional analysis of the z-slices maximum projections showed that *Hmga2* KO cells suffered from nuclear weakness, and they were also characterized by a reduced porosity compared to the wt cells that, instead, had an intact NL, which appeared as circular, thick, and definite (Fig. 3C).

The observed altered immunostaining of Lmnb1 was only dependent on the structure of the NL, because Lmnb1 expression was unchanged in *Hmga2* KO cells compared to wt cells (Additional file 5: Fig. S3A).

Noteworthy, no differences were observed between *Hmga2* wild-type and KO cells in the undifferentiated state (Additional file 5: Fig. S3B). This evidence indicated that the distortion of the NL in *Hmga2* KO cells occurred only when the cells were induced into EpiLCs, thus coinciding with the block of transition caused by *Hmga2* loss [16].

To confirm the data obtained in *Hmga2* KO cells, we examined the effects of *Hmga2* silencing in wt mouse ESCs, upon the induction of EpiLCs. *Hmga2* downregulation (see Additional file 5: Fig. S3C and D) strongly altered the structure of the NL, causing nuclear abnormalities similar to those observed in *Hmga2* KO cells (Fig. 3D, E).

**Fig. 4:**
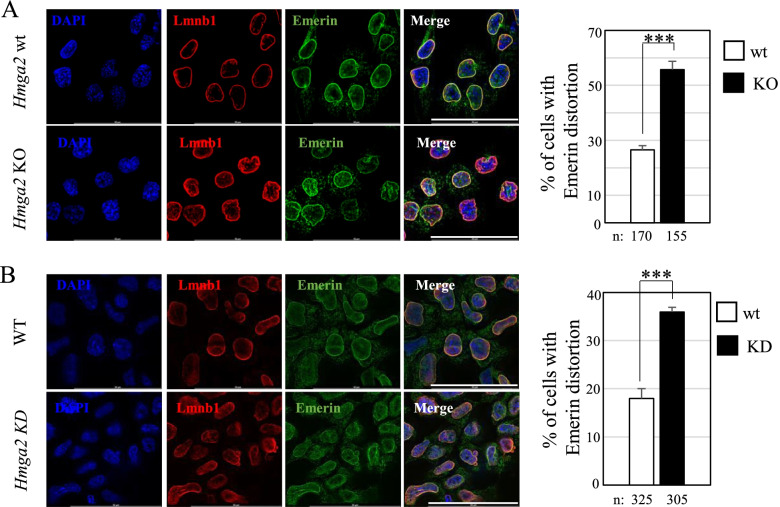
*Hmga2* silencing induced a phenotype identical to that observed in *Hmga2* KO cells. **A** Lmnb1 (red) and Emerin (green) immunofluorescence on *Hmga2* wt and KO cells (day 1 of EpiLC induction) showing the nuclear abnormalities typical of *Hmga2* KO cells (single z-plane, ROI 1024×1024, scale bar= 50μm). Quantification graphs obtained by counting >150 nuclei/condition. **B** Lmnb1 (red) and Emerin (green) immunofluorescence at day 3 after the induction of EpiLCs upon *Hmga2* silencing. Nuclear blebbing and enlarged nuclei were surrounded by both Lmnb1 and Emerin. DAPI (blue) was used to counterstain the nuclei. A single plane of the z-stack projection is shown. Scale bar= 50 μm. Quantification graphs obtained by counting >300 nuclei/condition. Error bars represent standard deviation. Statistical significance on three biological replicate experiments was determined using Student’s *t*-test (***p<0.001)

The nuclear lamina interposes between condensed chromatin and the inner nuclear membrane [26]. To examine the status of the inner nuclear membrane, we carried out the immunostaining with the anti-Emerin antibody in *Hmga2* wt and KO cells as well as in *Hmga2*-silenced EpiLCs, as this protein of the inner nuclear membrane is molecularly connected to the nuclear lamina. As reported in Fig. 4, the structural abnormalities detected using Lmnb1 antibody were also observed with Emerin antibody: the immunostaining revealed that the inner nuclear membrane of the *Hmga2* KO and *Hmga2*-silenced cells suffered from the distortion of the nuclear lamina. As for Lmnb1, no nuclear envelope defects were observed in Hmga2 KO cells compared to the wild-type cells in the undifferentiated conditions, confirming that the distortion occurred during the exit from the undifferentiated state (Additional file 6: Fig. S4).

Altogether, these data demonstrate that the absence of Hmga2 inside the nucleus prevents maintaining the proper structure of the nuclear lamina.

### Hmga2 loss affects the intranuclear distribution of H3K9me3

**Fig. 5:**
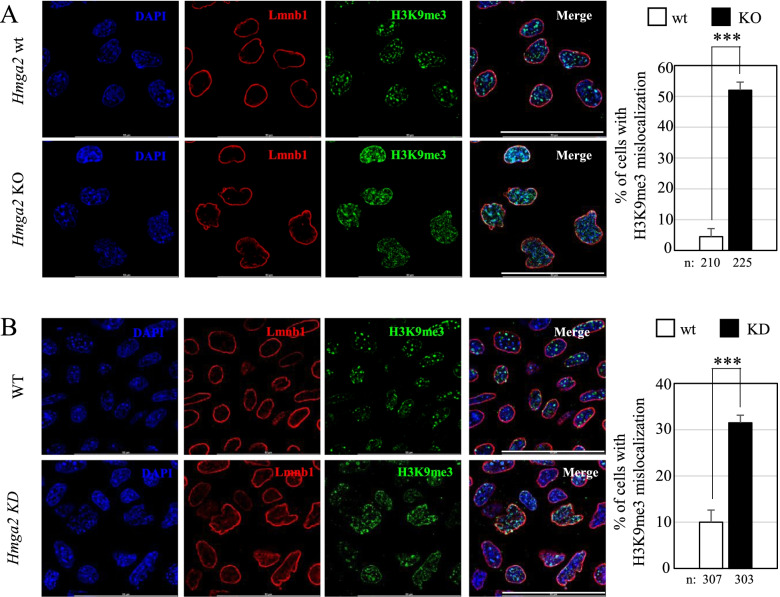
Mislocalization of H3K9me3 histone mark in *Hmga2* KO and KD cells upon EpiLC induction. **A** Immunoflurescence experiments on Hmga2 wt and KO cells at day 1 after the induction of EpiLCs of Lmnb1 (red) and H3K9me3 (green). **B** Lmnb1 (red) and H3K9me3 (green) immunofluorescence on *Hmga2* KD cells (day 3 after the induction). Non-silencing siRNA was used as control. In all immunofluorescence experiments, DAPI (blue) was used to counterstain the nuclei. All pictures are shown as single z-plane, ROI 1024×1024, scale bar= 50μm. Quantification graphs show the percentage of Hmga2 wt and KO cells with H3K9me3 mislocalization (*n*≥200 nuclei per condition) and the percentage of Hmga2 KD cells showing H3K9me3 mis-localization (*n*≥ 300 nuclei per condition). The count for Hmga2 wt and KO cells was performed at day 1 of differentiation, while the count for of Hmga2 KD cells was performed at day 3 of EpiLC transition, on three biological replicates for each condition. Error bars represent standard deviation. Statistical significance was determined using Student’s *t*-test (ns: not significant, ***p<0.001)

Chromatin associated with nuclear lamina is marked by heterochromatic histone modifications; thus, we investigated whether changes regarding the distribution and the abundance of these histone modifications occurred in *Hmga2* KO cells. First, we conducted immunofluorescence experiments to investigate H3K9me2/3 and H3K27me3 localization in *Hmga2* KO cells at day 1 of transition into EpiLCs. These experiments allowed us to detect a widespread mis-localization of H3K9me3 in differentiating *Hmga2* KO cells (Fig. 5A).

In detail, in the absence of *Hmga2*, H3K9me3 signal was diffuse inside the nucleus and not organized in intense dots at chromocenters (pericentric constitutive heterochromatin represented by DAPI-dense foci), as observed in the wild-type cells (Fig. 5A). H3K9me2 signal was mostly located close to nuclear envelope so its distribution in KO cells confirmed the distortion of nuclear lamina (Additional file 7: Fig. S5A). These results suggested that *Hmga2* loss affected H3K9me3 distribution, with effects on peripheral and internal heterochromatin. The distribution of H3K27me3 histone modification was not affected: *Hmga2* KO cells were characterized by small intense dots marked by H3K27me3 whose distribution was similar to that observed in wt cells (Additional file 7: Fig. S5B). A similar behavior was also observed in case of H3K4me3 and H3K27ac, which were similar in wt and KO cells (Additional file 7: Fig. S5C-D). In addition, we explored whether the increased levels of H3K27me3 and the slight accumulation of PRC2 subunits, Suz12 and Ezh2 (see Fig. 1), could have any role in the lamina-associated phenotype observed in Hmga2 KO cells. To this aim, we inhibited PRC2 by treating the cells with GSK126 for 72 h and inducing the transition towards EpiLCs for 24h. Although the treatment led to a clear decrease of H3K27me3, we observed the same alterations of the nuclear lamina as in mock treated cells (Additional file 8: Fig. S6).

*Hmga2* silencing in ESCs induced toward EpiLCs also confirmed the altered localization of H3K9me3 (Fig. 5B). It is worth noting that H3K9me3 and H3K27me3 accumulate within or close to nuclear blebs observed in *Hmga2* KO cells thus confirming the heterochromatin enrichment in nuclear abnormalities (HENA) observed in other experimental settings [27].

### Inter-TAD interactions and their association with nuclear lamina are altered in Hmga2 KO cells

The crosstalk between LADs with whole genome organization [25] and with the dynamics of TAD cliques during differentiation [20, 28] was clearly demonstrated in several conditions. On the other hand, in some cases, LAD borders are marked by H3K27me3 [24, 29], and the suppression of Ezh2, the catalytic subunits responsible for H3K27me3 deposition, prevents NL interaction with chromatin [27]. Thus, we hypothesized that defects in nuclear shape and heterochromatin mis-localization accompanying *Hmga2* suppression could be related to defects in 3D genome assembly.

To explore the effects of Hmga2 on genome 3D structure, we performed the Hi-C mapping in two independent clones of wt cells and in two clones of *Hmga2* KO cells, at day 3 after the induction of the transition into EpiLCs. After the filtering and mapping of Hi-C data of the four clones, we obtained 38,483,563; 55,451,307; 45,019,690; and 53,506,473 valid interactions, respectively (Additional file 9: Table S3).

**Fig. 6:**
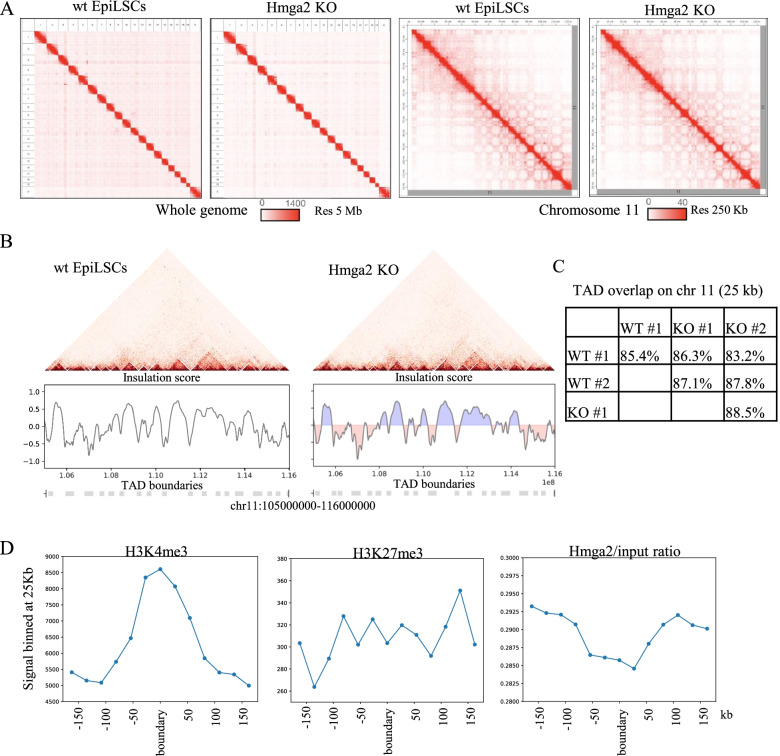
3D genome structure in wt and *Hmga2* KO cells. **A** Left: Genome-wide Hi-C data in wt and *Hmga2* KO conditions at 5Mb resolution, right: Hi-C data of chromosome 11 in both conditions at 250Kb resolution. **B** Hi-C maps in the region chr11:105000000-116000000, 25-Kb resolution. Insulation score and boundaries are reported below. **C** Overlap between boundaries (detected at 50kb resolution) in wt and *Hmga2* KO for chromosome 11. **D** Enrichment in WT of H3K4me3, H3K27me3, and Hmga2 (normalized) at TAD boundaries, shaded area represents the standard error of each point

The contact maps of all the chromosome and of an example (chromosome 11) are shown in Fig. 6A. Visual inspection of these maps indicate that the contact pattern is very similar in wt cells and in KO cells. To quantify this observation, we performed PCA analysis and identified A/B compartments genome-wide in wt and KO (Additional file 10: Fig. S7A) [30]. Comparison between compartments in different conditions reveals a general high correlation (genome-wide average Pearson *r*=0.96, Additional file 10: Fig. S7B), with few, short regions, typically located at the compartment periphery, exhibiting changes in compartment identity (Additional file 10: Fig. S7C, Additional file 11: Table S4). Next, we quantified the similarity of TAD boundaries [31, 32] in the different systems, called by their Insulation Score [33]. Such an analysis confirmed that TAD boundaries are similar in the four samples, with an overlap, in the case of chromosome 11, that was always close to 90% at 25 kb (see Fig. 6B and C and the “Methods” section), i.e., comparable to replicate similarity. Genome-wide analysis made with data at 25 kb indicated a global overlap close to 90%. Thus, the results indicated that the suppression of *Hmga2* does not alter TAD structure. The correlation of boundaries with epigenetic marks and with the binding of Hmga2 to chromatin confirmed that H3K4me3 is tightly associated with the boundaries [31], while no significant associations were observed in the case of H3K27me3 as previously reported. Interestingly, though, Hmga2 abundance on chromatin appears to be negatively associated with the boundaries (see Fig. 6D), highlighting a novel potential involvement of Hmga2 in TAD architecture.

**Fig. 7:**
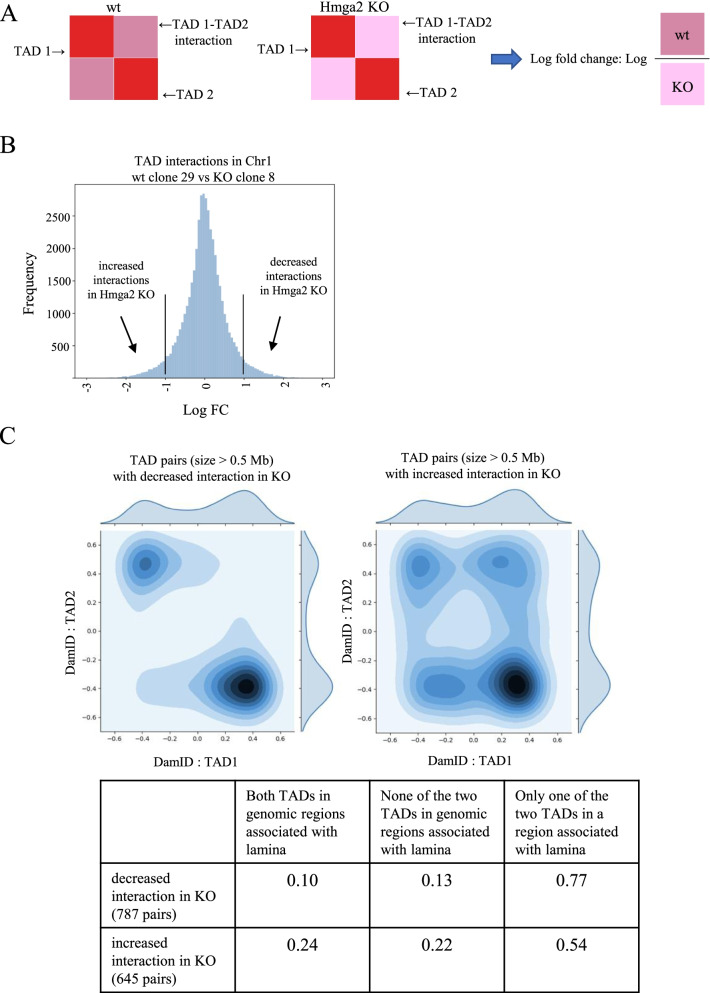
*Hmga2* KO alters inter-TAD interactions and their association with nuclear lamina. **A** The computational approach to analyze the effects of *Hmga2* KO on inter-TAD interactions was to calculate the Log fold change between the interactions between TADs in wt and *Hmga2* KO. **B** Fold changes between all TAD pairs were calculated for each chromosome; the graph reports the distribution of inter-TAD interactions in chromosome 1, showing that there are numerous interactions that are decreased or increased in *Hmga2* KO cells compared to wt cells. **C** Genome-wide enrichment of DamID signal [33] in TAD pairs exhibiting the most prominent interaction change

Next, we explored the effects of *Hmga2* KO on inter-TAD interactions. To this aim, we considered the average interaction between two given TADs and calculated the log ratio (or log fold-change, indicated as Log FC) between their interaction in wt cells vs Hmga2 KO cells. Figure 7A summarizes the computational approach to address this point.

The analysis of the log fold changes allowed us to identify the TAD pairs with most prominent differences between wt and KO cells, defined as those where LogFC >1 or <−1 (Fig. 7B, chromosome 1). In a significant number of cases, the interaction present in wt cells is decreased or increased by a factor of 2, highlighting that Hmga2, although not affecting TAD boundaries, has a role in the formation of chromatin 3D structure. Considering the phenotype of Hmga2 KO EpiLCs, and the demonstrated role of Lamins in the organization of global 3D genome structure [34], we asked whether there is any relationship between the changes we observed in the interactions among TADs and the chromatin regions preferentially associated with the nuclear lamina (DamID data taken from ref. [34]). To this aim, we selected all 787 pairs of TADs whose interaction is significantly decreased in Hmga2 KO cells (Log FC >1) and all 645 pairs that interact more in Hmga2 KO cells than in wt cells (LogFC<-1). The results show that, in most cases (77%) of TAD pairs with decreased interaction in KO cells, only one of the TADs is located in a region associated with Lamin, while only 23% of the pairs are either both associated with Lamin or not associated with Lamin (Fig. 7C). On the contrary, the TAD pairs showing an increased interaction in Hmga2 KO cells compared to wt cells are almost uniformly distributed (54% with only one of the two TADs associated with Lamin and 46% both associated with Lamin or not associated with Lamin). To check the robustness of these results, we repeated the above-described analysis using a random control system where we permuted (bootstrap) the boundary positions along the genome (Additional file 12: Fig. S8). Although TAD pairs were selected with the same criterion, no specific DamID enrichment pattern is observed in TAD pairs with increased or decreased interaction in KO as compared to the wt condition.

Finally, we checked that both types of TAD pairs exhibit analogous size distributions (Kolmogoroff-Smirnov test p-val > 0.05), so to exclude size-related effects. Taken together, our results highlight that the known role of Hmga2 in defining chromatin interaction with the lamina affects the establishment of TAD borders and inter-TAD interactions.

## Discussion

Hmga proteins have been studied for many years, in particular to address their role in cancer, where they are often overexpressed and were suggested to have a crucial role in tumorigenesis. However, the functions of these proteins are still not definitively addressed. In particular, their interaction with chromatin and the possible effects of this interaction have been explored in several studies. By using EpiSCs, where endogenous levels of Hmga2 are sufficiently high, we have studied the association of the protein to chromatin and we observed that it widely binds to chromatin. Only in a relatively small number of regions, an accumulation of Hmga2 was detectable, corresponding to very A/T-rich sequences. In any cases, we did not find any significant overlaps between the genes whose transcription is affected by Hmga2 suppression during differentiation and the genes whose transcription start sites are located within 100 kb from Hmga2 peaks. This suggests that the known effects of Hmga2 on the transcription of specific genes do not necessarily require a stable and continuous binding to chromatin, while Hmga2 interaction with chromatin appears to vary during the subsequent phases of the differentiation process. Thus, we cannot exclude that, in other cell types and in particular in cancer cells, Hmga2 could contribute to transcription regulation through the association with specific chromatin regions.

Our results are in good agreement with those obtained in ESCs overexpressing Hmga2 [22] that demonstrated the attitude of Hmga proteins to bind any chromatin region, with a particular preference for heterochromatic regions. In particular, the observation that Hmga2 is preferentially found in the chromatin regions associated with nuclear lamina allowed us to demonstrate that Hmga2 KO cells, upon the exit from naïve state, show evident distortions of the nuclear envelope. The latter is composed by nuclear membranes, crossed by numerous associated proteins [35], and the inner nuclear membrane is strictly connected to a filamentous meshwork of proteins, called nuclear lamina [26]. Nuclear lamina interacts with DNA, histones, transcription factors, and chromatin proteins [20], and, more in general, with large heterochromatin regions located at the nuclear periphery, defined lamina-associated domains (LADs) [36], marked by H3K9me2/me3 [29, 36]. We observed that the induction of EpiLCs from *Hmga2* KO and KD PSCs is characterized by a distortion of the nuclear lamina that results in evident abnormalities of the nuclear shape. This phenomenon is accompanied by a mis-localization of H3K9me3 within the nucleus. These observations suggest that the absence of Hmga2 hampers the establishment of the proper interactions between nuclear lamina and heterochromatin.

The absence of Hmga2 interferes with the deposition and/or the removal of H3K27me3, which marks some LAD boundaries [24, 29]. This histone modification is rapidly suppressed in wt cells soon after the exit from the naïve state, while continuing to be present at relatively high levels in *Hmga2* KO cells. This sustained presence of H3K27me3 could be the consequence of the persistence of the PRC2 activity, as suggested by the slightly increased levels of Suz12 and Ezh2 proteins in KO cells, likely due to post-transcriptional events. However, we did not find a significant relationship between genes regulated by PRC2 and genes whose expression was modified in Hmga2 KO cells, therefore it appears that the sustained deposition of H3K27me3 by PRC2 specifically concerns with nuclear lamina distortion and not with a general effect on bivalent promoters. The structure of Hmga2 suggests that it could be involved in the interaction of distant chromatin regions; indeed, the three AT-hooks domains present in this protein could bind distant DNA elements thus contributing to the 3D structure of the chromatin [10]. Accordingly, we observed that inter-TAD interactions are altered in the absence of Hmga2. This lack of interaction between cognate TADs and the wrong associations between TADs normally not interacting could result in a significant alteration of global 3D chromatin structure. The normal inter-TAD interactions that mostly suffer from the absence of Hmga2 are those involving one TAD endowed in Lamin-associated chromatin and the other one not associated with Lamins. This observation suggests the possibility that Hmga2 is involved in tethering euchromatic regions (TADs not directly interacting with Lamins) to the repressive environment where heterochromatic TADs are directly associated with the nuclear lamina. The increased deposition of H3K27me3, and the sustained expression of PRC2, could be interpreted as an attempt to keep repressed genes that, as a consequence of the absence of Hmga2, are localized in TADs which have lost their interaction with the heterochromatic TADs directly associated to the nuclear lamina. The compensatory effect of H3K27me3 was already observed in different experimental contexts, aimed at maintaining the silent state of altered heterochromatin [37, 38]. On the other hand, the TAD pairs showing an increased interaction in Hmga2 KO cells could result from inappropriate interactions between TADs, so explaining their uniform distribution among various chromatin regions, irrespective of their association with nuclear lamina.

The possible relationship between the loss of Hmga2 and the deposition of H3K27me3 by PRC2 is also suggested by a certain similarity of the phenotypes of the mice carrying mutations of *Hmga2* or of PRC2. Indeed, *Hmga2* KO mice display a pygmy phenotype, with reduced body weight and length [3] and this phenotype is exacerbated by the contemporary inactivation of the *Hmga1* gene [5]. An opposite phenotype was observed in transgenic mice where a mutant form of Hmga2 is constitutively expressed that show a giant phenotype [39]. Some rare human genetic diseases, associated with prenatal and postnatal overgrowth, are known as PRC2-related overgrowth syndromes (Weaver syndrome, Cohen–Gibson syndrome, and *SUZ12*-related overgrowth) [40, 41]. Accordingly, mice carrying a Weaver syndrome mutation, which show a reduction of H2K27me2-3, show overgrowth and organomegaly [42]. Although the similarity of these phenotypes could be a mere coincidence, it probably deserves further studies.

The findings reported herein could also contribute to the understanding of the role of Hmga2 in cancer. It is evident that Hmga2 expression is increased in many different types of tumors and in some cases these tumors appear to be Hmga2 addicted, as its suppression results in a dramatic change in cancer behavior and aggressiveness [15]. Most results available suggest that these phenomena are the consequence of Hmga2-induced changes in gene expression. To address whether these changes are due to the direct regulation of transcription by Hmga2 and also to a general role of this protein in the 3D genome organization, as it emerges from our results, deserves further studies.

## Conclusions

Here, we have demonstrated that the loss of Hmga2 leads to an inappropriate persistence of H3K27me3 histone mark after the exit from the naïve state of PSCs. Moreover, the absence of Hmga2 has a dramatic effect on the structure of NL and on the proper localization of the heterochromatin mark H3K9me3 within the nucleus. These nuclear alterations found in Hmga2 KO cells influence the inter-TAD interactions indicating that Hmga2 is required for the establishment of the proper 3D structure of the chromatin.

## Methods

### Cell culture, transfection, EpiLC and EpiSC generation, and SFEB differentiation

*Hmga2* wt and KO induced PSCs (iPSCs) were generated as previously described [16]. The cells were grown on mitotically inactivated fibroblast feeder layers and expanded in the following iPSC medium: GMEM supplemented with 2 mM glutamine, 100 U/mL penicillin/streptomycin, 1 mM sodium pyruvate, 1X non-essential amino acids, 0.1 mM β-mercaptoethanol (all from Invitrogen), 15% FBS (Hyclone), 10^3^ U/ml leukaemia inhibitory factor (EMD Millipore), and 2i (MEK and GSK3 inhibitors; 1 μΜ PD0325901 and 3 μΜ CHIR-99021, both from Selleckchem). Two clones of *Hmga2* wt iPSCs and two clones of *Hmga2* KO iPSCs were used throughout this study.

Mouse ESCs (E14Tg2a, Bay Genomics) were expanded on gelatin-coated plates in the following ESC medium: GMEM supplemented with 2 mM glutamine, 100 U/mL penicillin/streptomycin, 1 mM sodium pyruvate, 1X non-essential amino acids, 0.1 mM β-mercaptoethanol, 10% FBS, and 10^3^ U/ml leukaemia inhibitory factor.

iPSCs and ESCs were induced into EpiLCs according to the following protocol: 2×10^4^ cells/cm^2^ were plated on plates coated with Geltrex™ LDEV-Free Reduced Growth Factor Basement Membrane Matrix (Gibco) in EpiLC medium (DMEMF12/Neurobasal medium 1:1 (Gibco), 0.5% N2 supplement (Thermo Fisher), 1% B27 supplement (Thermo Fisher), BSA (Sigma Aldrich), 2 mM glutamine (Invitrogen), 20 ng/mL Activin A (R&D Systems), and 12 ng/mL bFGF (Invitrogen). For RNA and protein extraction and immunofluorescence, the cells were harvested at different time points as specified in the figures after 1, 2, and 3 days of EpiLC induction. For the treatment with PRC2 inhibitor, iPSCs were plated on gelatin-coated plates and grown in the presence or in the absence of 10 μM GSK126 (S7061, Selleckchem) for 72 h. DMSO was used as vehicle control. Then, the cells were differentiated into EpiLCs according to the protocol described above.

*Hmga2* wt and KO iPSCs were differentiated into SFEBs using the protocol in ref. [43]. Briefly, 1×10^6^ cells were plated in 100-mm Petri dishes in the following differentiation medium: Glasgow minimum essential medium supplemented with 2 mM glutamine, 1 mM sodium pyruvate, 1X non-essential amino acids, 0.1 mM β-mercaptoethanol, and 10% KO serum replacement (ThermoFisher). The aggregates were harvested for protein extraction at 2, 3, and 4 days of differentiation.

To induce *Hmga2* knockdown, 2×10^4^ cells/cm^2^ ESCs were plated in EpiLC medium. Twenty-four hours after plating (at 1 day of EpiLC induction), the cells were transfected with a pool of three different small interference RNAs (siRNAs, Invitrogen) directed against *Hmga2* transcript (si*Hmga2* MSS274854, si*Hmga2* MSS274855, si*Hmga2* MSS274856) using Lipofectamine 2000 (Invitrogen), according to the manufacturer’s protocol. The cells were harvested at day 3 for protein and RNA extraction as well as for immunofluorescence experiments.

For ChIP-seq experiments *Hmga2* wt and KO iPSCs were induced into EpiSCs by using the protocol in ref. [44]. In detail, 3×10^3^/cm^2^ cells were grown in a medium containing N2 and B27 supplements (Thermo Scientific) complemented with 20 ng/mL Activin A (R&D Systems) and 12 ng/mL bFGF (Invitrogen). The cells were cultured in this medium for 3 days dissociated with dispase (Gibco) and cultured on ™ LDEV-Free Reduced Growth Factor Basement Membrane Matrix (Gibco)-coated plates. The cells were expanded repeating this procedure every 2–3 days for 2 weeks and then collected for ChIP-seq experiments.

### Protein extraction and western blotting

For protein extraction, the cells were washed twice with ice-cold 1X phosphate saline buffer (1X PBS) and lysed with RIPA Lysis and Extraction Buffer (Thermo Scientific), complemented with protease (Sigma-Aldrich) and phosphatase inhibitors, according to the manufacturer’s protocol. After lysis, the samples were sonicated with Minisonix XL 2000 and centrifugated at 14000×g for 15 min to isolate total nuclear proteins. Bradford Protein assay (Bradford Reagent, Bio-Rad) was used to determine the concentration of total proteins. Fifteen micrograms of total protein was loaded in each lane. For western blotting experiments, the following antibodies were used: anti-Hmga2 #5269 (Cell Signaling) anti-H3K27me3 #3915S, anti-H3 (clone 0301) #39064, anti-H3K4me3 #61379, anti-H3K9me2 #39239, anti-H3K9me3 #39161 (all from Active Motif), anti-H3K27ac ab4729 (Abcam), anti-H3K27me2 ab26684, anti-Lmnb1 D4Q4Z (Cell Signalling), and anti-Gapdh sc-32233 (Santa Cruz Biotechnology). The secondary antibodies used in this study were goat anti-rabbit IgG (HeL), HRP conjugate GtxRb-003-DHRPX (ImmunoReagents) or goat anti-mouse IgG (HeL), and HRP conjugate GtxMu-003-DHRPX. Clarity TM western ECL substrate (170-5060, Bio-Rad) was used for signals detection by enhanced chemiluminescence. Western blotting images were acquired with ChemiDoc MP Imaging System (Bio-Rad). Band intensities were quantified using Image J software. The experiments were carried out in triplicate.

### RNA extraction, reverse transcription, real-time qPCR, and RNA-sequencing

Total RNA was extracted using Tri-Sure (Bioline), according to the manufacturer’s protocol. Full-length first cDNA strand was synthetized by RevertAid Reverse Transcriptase (Thermo Scientific). RNA concentration and purity was determined using Nanodrop 2000c (Thermo Scientific). qRT-PCR was run on QuantStudio 7 Flex Real Time PCR System using Fast SYBR Green PCR Master Mix (Thermo Fisher Scientific). The sequence of the primers used to determine the expression of *Hmga2* transcript was as follow: 5′-AAAACGGCCAAGAGGCAGAC-3′, 5′-ATGTCTCTTCAGTCTCCTGAGCA-3′. The expression of glyceraldehyde-3-phosphate dehydrogenase (*Gapdh*, 5′-GTATGACTCCACTCACGGCAAA-3′, 5′-TTCCCATTCTCGGCCTTG-3′) was used as internal control. Gene expression was evaluated by ΔΔCt method. The experiments were carried out in triplicate. For RNA-seq and analysis, total RNA was extracted from *Hmga2* wt and KO cells induced into EpiLCs as described above and sequenced using the pipeline reported in our previous study [45].

Next-generation sequencing experiments were performed by Genomix4life S.R.L. (Baronissi, Salerno, Italy). RNA concentrations were determined by using NanoDropOne spectrophotometer (Thermo Fisher) and its quality assessed with the TapeStation 4200 (Agilent Technologies). Indexed libraries were prepared from 500 ng of purified RNA with TruSeq Stranded totalRNA Sample Prep Kit (Illumina) according to the manufacturer’s instructions. Libraries were quantified using the TapeStation 4200 (Agilent Technologies) and Qubit fluorometer (Invitrogen Co.), then pooled such that each index-tagged sample was present in equimolar amounts. The pooled samples were subject to cluster generation and sequencing using an Illumina NovaSeq6000 System (Illumina) in a 2×75 paired-end format.

Sequencing reads were processed using “nf-core’s” [46] “rnaseq” pipeline (ver.: 1.2.2) run within Dockers [47] container through Nextflow process scheduler [48]. In short, raw reads were evaluated with FastQC, the 5′ and 3′ adapters were trimmed using Trim Galore, and genome/ribosomal RNA sequences were discarded with BBSplit/SortMeRN. Filtered reads were aligned to Ensembl’s GRCm38 reference genome from Amazon’s iGenomes database using STAR sort, indexed with SAMtools, duplicate marked using Picard and gene/transcript quantified applying Salmon/StringTie. Finally, gene expression levels were compared between cell lines using DESeq2. Statistical analysis performed using the computing environment R [49].

Total RNA isolated from three independent biological replicates was used for RNA-seq experiments.

### Chromatin-immunoprecipitation (ChIP)-sequencing

ChIP experiments were performed on EpiSCs. Briefly, the cells were crosslinked with 1% formaldehyde for 10 min at room temperature and next quenched by addition of glycine (125 mM). The samples were sonicated to obtain fragments with a length of 200–500bp. Chromatin extracts were incubated with anti-Hmga2 antibody (Cell Signaling, #5269, 1:100). Appropriate IgG (Abcam) were used as a negative control, while the supernatant without antibody was used as input control. Oligonucleotide pairs used are the following:

- Peak 7040: for-TAGATCTGTGGGGGTCCAGG, rev-CTGGCCCTGTTGTAACCAGT
- Peak 4169: for-GGGGAGAGCATGCCTTTGAT, rev- TGGAGAGATGGCTGGACTCA
- Peak 10319: for-ACATTTACTGTTCCTCACAGCCT, rev-ACGTATGAATCCCTTCGCCA
- Peak 10994: for-GTCTTGTCTAAACCCAGGTGGA, rev-GTCTGGAATGGACACGGTGA
- Peak 15807: for-GAGCCCACCTACGTCAATCA, rev-ACCGAGCAAATTCCTCCCTG
- miR-23a: for-CTTGGCTCCCTGTACCTGTC, rev-TTACCTTCTCAGGCCACCCT

ChIP-seq library was prepared using ChIP-Seq Sample Prep Kit (Illumina) and sequenced on the Illumina HiScanSQ Platform, as in ref. [50]. The obtained reads were mapped to the mouse genome (mm9 assembly). Sequencing reads were processed using “nf-core’s” [46] “chipseq” pipeline (ver.: 1.2.2) run within Docker [47] container through Nextflow process scheduler. In short, raw reads were evaluated with FastQC [48], and the 5′ and 3′ adapters were trimmed using Trim Galore. Then, trimmed reads were aligned against Ensembl’s GRCm38 reference genome from Amazon’s iGenomes database with BWA and filtered out from ambiguous reads using Picard, SAMtools, BAMtools, and Pysam. Afterwards, the peaks were called with MACS2, providing input samples from the chip-seq experiment as a control, and Hmga2 enrichments were annotated using HOMER. Finally, Hmga2 peaks were compared between cell lines using DESeq2. Statistical analysis was performed using the computing environment R [49]. H3K4me1, H3K27ac, and H3K27me3 ChIP-seq data are from ref. [19].

### Immunofluorescence

Immunofluorescence experiments on undifferentiated iPSCs were conducted plating the cells on gelatin-coated Nunc ^TM^ Lab-Tek ^TM^ chamber slides (Thermo Scientific). For immunofluorescence experiments on EpiLCs, the cells were plated on coverslip coated with Geltrex™ LDEV-Free Reduced Growth Factor Basement Membrane Matrix (Gibco) and induced into EpiLCs. The cells were harvested at day 1 or 3 of EpiLC transition, washed with 1X PBS and fixed with 4% paraformaldehyde for 10 min at room temperature. Next, they were permeabilized with 0.2% Triton-X100 (PanReac Applichem) for ten minutes and incubated with the blocking solution (10% FBS, 1%BSA, 1X PBS) for 30 minutes at room temperature. For antigen detection, the cells were incubated with anti-H3K27me3 #3915S, anti-H3K9me2 #39239, anti-H3K9me3 #39161 anti-H3K27me2 ab26684 antibodies (all from Active Motif), anti-H3K27ac ab4729 (Abcam), anti-Lmnb1 (B-10) sc-374015 (Santa Cruz Biotechnology), and anti-Emerin (D3B9G) XP ® Rabbit mAB#30853 (Cell Signalling) overnight at 4°C. The day after, the coverslips were washed with 1X PBS and incubated with the secondary antibody donkey anti-Rabbit IgG (H + L), Alexa Fluor™ 488, or goat anti-Rabbit IgG (H+L) Alexa Fluor™ 594 (Invitrogen, USA) for 1 h at room temperature. After washes, the cells were stained with DAPI (268298, Calbiochem) for 5 min at room temperature. Images were captured using Leica Thunder Imaging System (Leica Microsystems) equipped with a LEICA DFC9000 GTC camera, lumencor fluorescence LED light source, and 100× oil immersion objective was used to acquire Z-slice images. Small volume computational clearing was used to remove the background signal derived from out-of-focus blur.

### Hi-C sequencing

*Hmga2* wt and KO cells were induced into EpiLCs and at day 3 were washed with 1X PBS and trypsinized for 5 min at 37°C. The cells were centrifuged for 10 min at 450×g at room temperature and the pellet gentled resuspended in EpiLC fresh medium. Next, the cells were cross-linked with 1% formaldehyde for 10 min at room temperature and incubated with 3M Tris pH 7.5 for 5 min at room temperature and then on ice for 15 min to quench the formaldehyde. The cells were centrifugated for 10 min at 800×g at 4° C, the pellet was resuspended in cold 1X PBS, and the cell density was determined. Hi-C sequencing was performed by Genomix4life S.R.L. (Baronissi, Salerno, Italy) [51]. Indexed libraries were prepared from 2×10^6^ cells, with EpiTect® Hi-C (Qiagen) according to the manufacturer’s instructions. Libraries were quantified using the TapeStation 4200 (Agilent Technologies) and Invitrogen Qubit fluorometer (Thermo Fisher Scientific), then pooled such that each index-tagged sample was present in equimolar amounts, with final concentration of the pooled samples of 2nM. The pooled samples were subject to cluster generation and sequencing using an Illumina NextSeq 550 System (Illumina) in a 2×150 paired-end format. Paired-end reads were aligned to the NCBI reference sequence (mm10) using the read-analysis for Hi-C reads published [52] pipeline named Hi-C-Pro. Two different clones of *Hmga2* wt cells and two different clones of *Hmga2* KO cells were used for Hi-C experiments.

### Hi-C data analysis

Hi-C data were analyzed using the platform *Juicer* [53]. For most analyses, we used Hi-C maps binned at 25kb resolution and KR normalized [54]. TAD boundaries were called using the insulation score approach [33], with a standard insulation window having a 500Kb × 500Kb size. We checked the robustness of the results by applying the procedure on Hi-C data binned at 50kb and returned, as expected, similar boundaries. Overlapping boundaries in wt and Hmga2 KO are defined as boundaries which have a genomic distance less than a tolerance threshold set to 3 bins (75kb at 25kb resolution and 150kb at 50kb resolution), i.e., if two boundaries are within this threshold they are considered overlapping, otherwise not [55]. TADs are defined as genomic regions between two consecutive boundaries. Interaction between different TADs (say TAD1 and TAD2, Fig. 7a) is defined as the average Hi-C score: ${X}_{1,2}=\frac{\sum_{i,j}{x}_{i,j}}{a1\ast a2}$ where *a*1 (*a*2) is the size of TAD1 (TAD2), *x*_*i*, *j*_ is the Hi-C score between bin *i* and bin *j*, *i* (*j*) runs over the entries of the Hi-C matrix corresponding to TAD1 (TAD2) coordinates. Taking as reference the TAD boundaries called in wt conditions, fold change is the ratio between the average Hi-C scores $F{C}_{1,2}={X}_{1,2}^{wt}/{X}_{1,2}^{Hgma2 KO}$. In order to have Log FC values symmetrically distributed around 0, the distribution is shifted so to have a null median (Fig. 7B). In general, TAD pairs with low (high) Log FC are classified as more (less) interacting in Hmga2 KO than wt condition. In addition, TAD pairs are classified according to their “TAD distance”, i.e., the number of TADs separating two TADs in the pair. For each distance, we consider the two most/least interacting pairs in KO, so to equally consider different genomic distances. Finally, we consider TADs having a size higher than 500kb. For each TAD, we evaluated lamina association level by measuring the average DamID signal (binned at 100kb, from ref. [34]) contained in the TAD. As DamID was available for mm9 assembly, we employed the *liftover* tool to remap our TAD boundaries from mm10 to mm9 assembly in this analysis. Average DamID levels for TAD pairs are then represented on a 2D plane (Fig. 7c). As control case, we generated control boundaries configurations by randomly permuting real boundaries on each chromosome, so to conserve the same TAD size distribution. Then, we calculated on this new arrangement of randomly located TADs the average DamID level, as described above. Enrichment profiles of H3K4me3, H3K27me3 and Hmga2 marks at wt TAD boundaries are computed by aggregating the average signal (binned at 25kb) of a 300-kb interval centered around each boundary. For each point, average and standard error is estimated over the genome-wide boundary distribution.

PCA analysis was performed on Hi-C data at 100 Kb resolution using the function *eigenvector* in *Juicer*, as described in [52]. Then, the first eigenvector was correlated with GC content (https://genome.ucsc.edu/cgi-bin/hgTables) to call A/B compartments [54–57]. Finally, genomic regions exhibiting changes in A/B compartment membership from wt to KO condition were simply defined according to the change in the eigenvector signal, i.e., regions marked as A in WT and B in KO (A→B) and, vice-versa, as B in WT and A in KO (B→A), as shown in Additional file 10: Fig. S7C.

## Supplementary Information

**Additional file 1: Figure S1.** Hmga2 western blot in wt and KO cells; quantification of western blot of Fig. 1A. (A) western blot analysis of wt and KO cells with Hmga2 ab; Gapdh was used as loading control. (B) The data in the graphs represent the mean ± SD (n=3 biological replicates) of H3K27me3, H3K4me3, H3K9me2, H3K9me3 and H3K27ac against total H3. Student’s t-test, two tailed (ns: not significant, *p<0.05).

**Additional file 2: Table S1.** Expression changes at 24h after the induction of differentiation in Hmga2 wt and KO cells.

**Additional file 3: Table S2.** Hmga2 ChIP-seq data.

**Additional file 4: Figure S2.** Histone marks at Hmga2 peaks in wt and Hmga2 KD EpiSCs. Chromatin from EpiSCs transfected with control (WT) and Hmga2 targeting siRNAs (KD) was immunoprecipitated with IgG or anti-H3K4me1 or anti-H3K27ac antibodies. Immunoprecipitated DNA was amplified by using oligo pairs annealing to the indicated Hmga2 peaks, which were associated by ChIP-seq with H3K4me1 and/or H3K27ac peaks. A region overlapping the miR-23a gene enhancer was used as a control; Hmga2 peak 10319, not associated with histone modifications, was used as a negative control.

**Additional file 5: Figure S3.** Lmnb1 abnormalities occur during the transition from the naïve state to the primed state of pluripotency. (A) Western blotting image showing Lmnb1 expression in Hmga2 wt and KO cells at day 3 after the induction of EpiLCs. Two different clones of PSCs were induced into EpiLCs for each condition. Western blotting quantification revealed no differences in terms of Lmnb1 expression between Hmga2 wild type and knock-out cells. The data in the graphs represent the mean ± SD of the Lmnb1 signal against Gapdh (n=3 biological replicates). ns: not significant (Student’s t-test, two tailed). (B) Lmnb1 immunofluorescence (red) on undifferentiated Hmga2 wt and KO cells showing no structural abnormalities of the nuclei. DAPI (blue) was used to counterstain the nuclei. A single plane of z-stack projection is shown. Scale bar = 50 μm. The data in the graphs represent the mean ± SD of the Lmnb1 signal against Gapdh (n=3 biological replicates). ns: not significant (Student’s t-test, two tailed). (C) q-PCR analysis to evaluate the levels of Hmga2 mRNA in EpiLCs upon transfection of siRNA. Non-silencing siRNA was used as control. The data in the graphs are reported as mean ± SD of relative values (fold changes) obtained normalizing the data of Hmga2 silencing with those of non-silencing control siRNA. **p<0.01, Student’s t-test, two tailed. (D) Western blotting image showing Hmga2 protein expression levels in EpiLCs upon transfection of non-silencing control siRNA and Hmga2 siRNA, respectively.

**Additional file 6: Figure S4.** Absence of nuclear alterations in Hmga2 KO undifferentiated cells. Lmnb1 (red) and Emerin (green) immunofluorescence on undifferentiated Hmga2 wt and KO cells showing the absence of nuclear abnormalities. DAPI (blue) was used to counterstain the nuclei. Maximum projection of z-slices (ROI 1024x1024) is shown. Scale bar = 50 μm.

**Additional file 7: Figure S5.** Hmga2 wt and KO cells showed a similar localization of several histone H3 modifications upon the induction of EpiLCs. Immunoflurescence experiments on Hmga2 wt and KO cells at day 1 after the induction of EpiLCs. Lmnb1 (red) and H3K4me3, H3K9me2, H3K27me3 and H3K27ac (green) as indicated. Scale bars=50 μm. Quantification graphs obtained by counting >200 nuclei/condition. Error bars represent standard deviation. Statistical significance on three biological replicate experiments was determined using the student’s t-test, ns: not significant).

**Additional file 8: Figure S6.** Treatment of wt and KO cells with PRC2 inhibitor and its effect on nuclear lamina. (A) Western blotting image showing the level of H3K27me3 histone modifications in Hmga2 wt and KO PSCs treated or not with PRC2 inhibitor at day 0 and at day 1 after the induction of the transition into EpiLCs. The levels of the histone H3 were used as control. (B) Immunoflurescence experiments on Hmga2 wt and KO cells treated or not with PRC2 inhibitor at day 1 after the induction of EpiLCs showing the nuclear lamina phenotype. All pictures are shown as single z-plane, ROI 1024x1024, scale bar=50μm. Quantification graph showing the percentage of cells with nuclear lamina distortion in Hmga2 wt and KO cells treated or not with PRC2 inhibitor (n≤100 nuclei per condition). The count was performed at day 1 of EpiLC transition on three biological replicates. Error bars represent standard deviation. Statistical significance was determined using the student’s t-test (ns: not significant).

**Additional file 9: Table S3.** Hi-C data summary.

**Additional file 10: Figure S7.** Identification of A/B compartment. (A) 1^st^ eigenvector from the PCA analysis is shown in wt and KO cells. GC content (reported below) is used to call A/B compartment. All signals are binned at 100kb. (B) Pearson correlation between the 1^st^ eigenvector in wt and KO for each chromosome. Dashed line represents the average value. (C) Examples of genomic regions exhibiting changes in A/B compartment membership from wt to KO condition.

**Additional file 11: Table S4.** Genomic coordinates of A->B and B->A compartment changes observed in Hmga2 KO cells.

**Additional file 12: Figure S8.** Simulated comparison of inter-TAD interactions. The analysis reported in panel C of Fig. 7 was repeated using a random control system where the boundary positions along the genome were permuted.

**Additional file 13.** Uncropped blots.

**Additional file 14.** Data.

## Data Availability

All data generated or analyzed during this study are included in this published article, its supplementary information files, and publicly available repositories. RNAseq, CHIPseq, and Hi-C data are available at GEO (GSE200673). Uncropped blots and supporting data values are in the Additional files 13 and 14, respectively. Publicly available datasets analyzed in this study are accessible at GEO with the reference ID GSE17051 for DamID in mouse ESCs [20], GSM1382217 and GSM1382219 for chromatin markers H3K4me1 and H3K27ac [19], GSE74330 for PRC2 ChIP-seq [21], and GSE58101 for MNase-seq [23].

## Abbreviations

ChIP: Chromatin immunoprecipitation
ESCs: Embryonic stem cells
EpiLCs: Epiblast-like stem cells
EpiSCs: Epiblast stem cells
NL: Nuclear lamina
LADs: Lamin-associated domains
PSCs: Luripotent stem cells
qPCR: Quantitative real time PCR
TADs: Topologically associated domains
