# Supplementary material for: Hmga2 protein loss alters nuclear envelope and 3D chromatin structure
Source: BMC Biol. 2022 Aug 2;20:171. doi: 10.1186/s12915-022-01375-3 (PMC9344646; doi:10.1186/s12915-022-01375-3)
Supplement: Supplementary file 1 — Additional file 1: Figure S1. Hmga2 western blot in wt and KO cells; quantification of western blot of Fig. 1A. (A) western blot analysis of wt and KO cells with Hmga2 ab; Gapdh was used as loading control. (B) The data in the graphs represent the mean ± SD (n=3 biological replicates) of H3K27me3, H3K4me3, H3K9me2, H3K9me3 and H3K27ac against total H3. Student’s t-test, two tailed (ns: not significant, *p<0.05). [file 12915_2022_1375_MOESM1_ESM.pptx]

## Slide 1
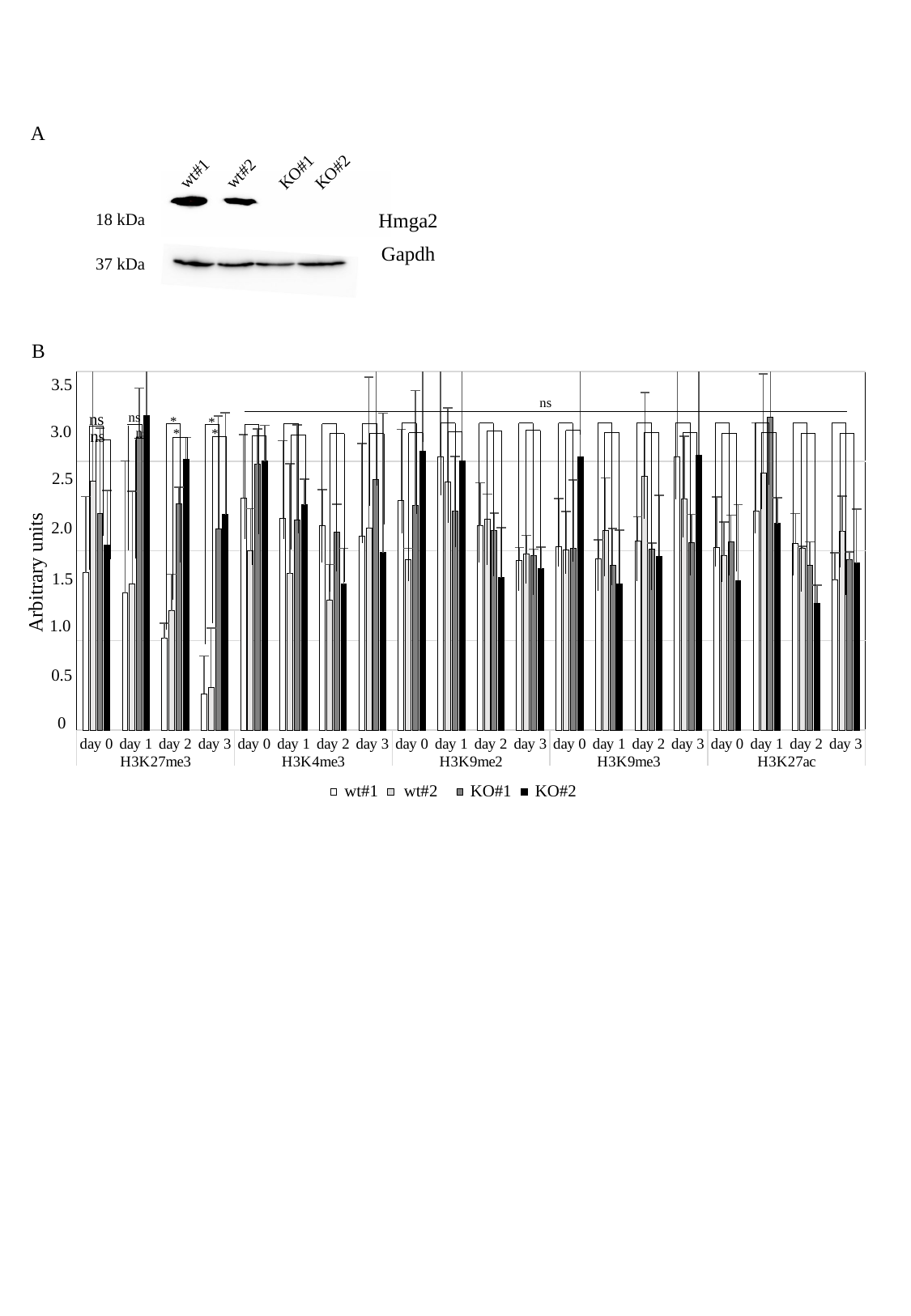

A
KO#1
KO#2
wt#1
wt#2
Hmga2
18 kDa
Gapdh
37 kDa
B
### Chart
| Category | cl28wt | cl29wt | cl6KO | cl8KO |
|---|---|---|---|---|
| day 0 | 0.8767731560771992 | 1.3894790268690638 | 1.208206990441637 | 1.0331829416989617 |
| day 1 | 0.7639997393311933 | 0.8151782823212144 | 1.6313781875984947 | 1.7543001755695051 |
| day 2 | 0.5111472068901731 | 0.6644153252652255 | 1.2630641817614834 | 1.5123924162605775 |
| day 3 | 0.20298135421863026 | 0.23927300504490426 | 1.122951813465339 | 1.2016838468918514 |
| day 0 | 1.2957466759273004 | 1.0 | 1.4814386584293242 | 1.5 |
| day 1 | 1.182246108807449 | 0.8726356994029177 | 1.1724922235844188 | 1.2558224690828859 |
| day 2 | 1.1414813835331814 | 0.7254398653064027 | 1.1039700914534223 | 0.8135266602120685 |
| day 3 | 1.0818575035897924 | 1.1255739478858284 | 1.3991668088300788 | 0.9895476287931775 |
| day 0 | 1.282033238246896 | 0.9524974560410399 | 1.2510729433617473 | 1.5558853148745786 |
| day 1 | 1.5217723007327926 | 1.384882432411014 | 1.2192673000247083 | 1.4992452122871491 |
| day 2 | 1.138735389608277 | 1.1740604179002272 | 1.1125330240859255 | 0.8509253542517113 |
| day 3 | 0.9480738468594311 | 0.9813462401244246 | 0.9717889620223251 | 0.8989155316982629 |
| day 0 | 1.0243497186718191 | 1.00704067765991 | 1.0118833905588878 | 1.5258501243015081 |
| day 1 | 0.954917639130124 | 1.1146829202375905 | 0.9194178576227902 | 0.8160444527917449 |
| day 2 | 1.0548529456204596 | 1.4163404151493026 | 1.0107283305058326 | 0.9690447394872616 |
| day 3 | 1.523458751997077 | 1.287955289641716 | 1.0466811831920504 | 1.5335091834033256 |
| day 0 | 1.0188693356681988 | 0.9729954756169028 | 1.0514601378305715 | 0.8331244021137859 |
| day 1 | 1.2207829774612766 | 1.4353786605485002 | 1.746693496151022 | 1.1519058727656324 |
| day 2 | 1.0429999435104975 | 1.0131772230466227 | 0.9211217618368784 | 0.7077250939539379 |
| day 3 | 0.83732074193412 | 1.1083957240387283 | 0.9492436983230274 | 0.9321654982665871 |3.5
ns
ns
ns
*
*
3.0
ns
*
*
ns
2.5
2.0
Arbitrary units
1.5
1.0
0.5
0
wt#1
wt#2
KO#1
KO#2
