# Supplementary figures and images for: Hmga2 protein loss alters nuclear envelope and 3D chromatin structure
Source: BMC Biol. 2022 Aug 2;20:171. doi: 10.1186/s12915-022-01375-3 (PMC9344646; doi:10.1186/s12915-022-01375-3)

## Slide 1
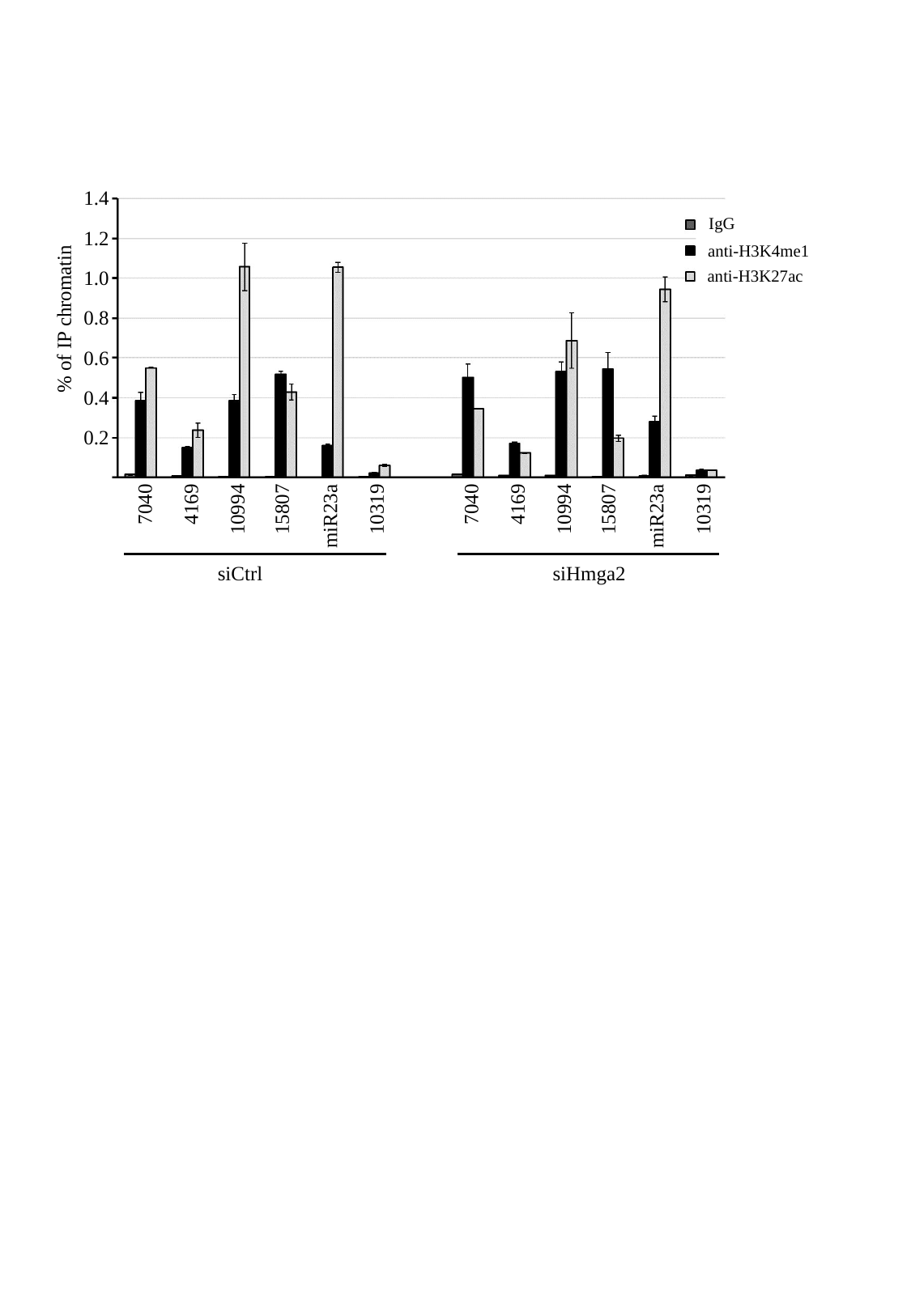

1.4
IgG
anti-H3K4me1
anti-H3K27ac
1.2
1.0
0.8
% of IP chromatin
0.6
0.4
0.2
7040
4169
7040
4169
10994
10319
10994
15807
15807
10319
miR23a
miR23a
siCtrl
siHmga2

Supplement: Supplementary file 4 — Additional file 4: Figure S2. Histone marks at Hmga2 peaks in wt and Hmga2 KD EpiSCs. Chromatin from EpiSCs transfected with control (WT) and Hmga2 targeting siRNAs (KD) was immunoprecipitated with IgG or anti-H3K4me1 or anti-H3K27ac antibodies. Immunoprecipitated DNA was amplified by using oligo pairs annealing to the indicated Hmga2 peaks, which were associated by ChIP-seq with H3K4me1 and/or H3K27ac peaks. A region overlapping the miR-23a gene enhancer was used as a control; Hmga2 peak 10319, not associated with histone modifications, was used as a negative control. [file 12915_2022_1375_MOESM4_ESM.pptx]

## Slide 1
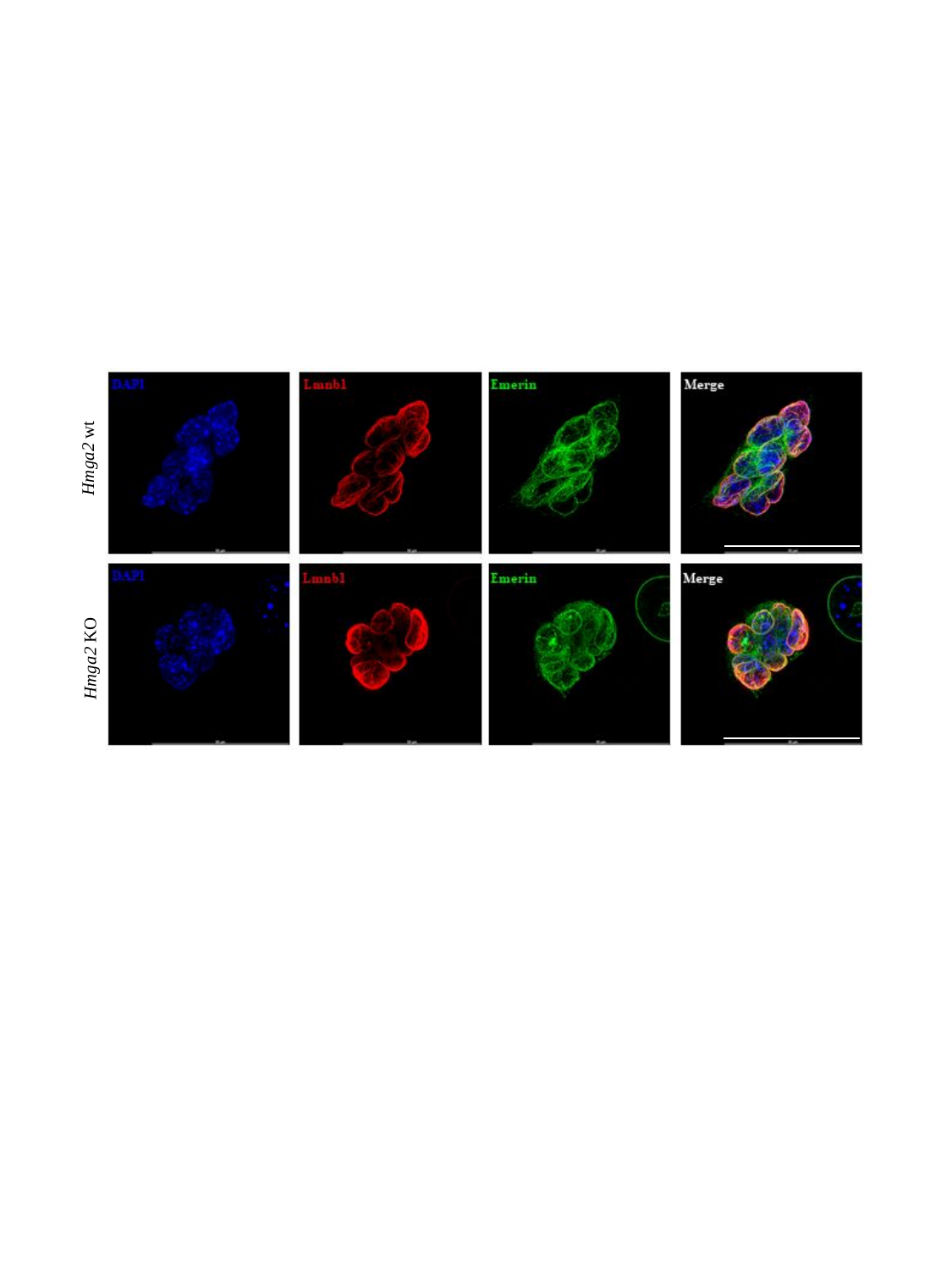

Hmga2 wt
Hmga2 KO

Supplement: Supplementary file 6 — Additional file 6: Figure S4. Absence of nuclear alterations in Hmga2 KO undifferentiated cells. Lmnb1 (red) and Emerin (green) immunofluorescence on undifferentiated Hmga2 wt and KO cells showing the absence of nuclear abnormalities. DAPI (blue) was used to counterstain the nuclei. Maximum projection of z-slices (ROI 1024x1024) is shown. Scale bar = 50 μm. [file 12915_2022_1375_MOESM6_ESM.pptx]

## Slide 1
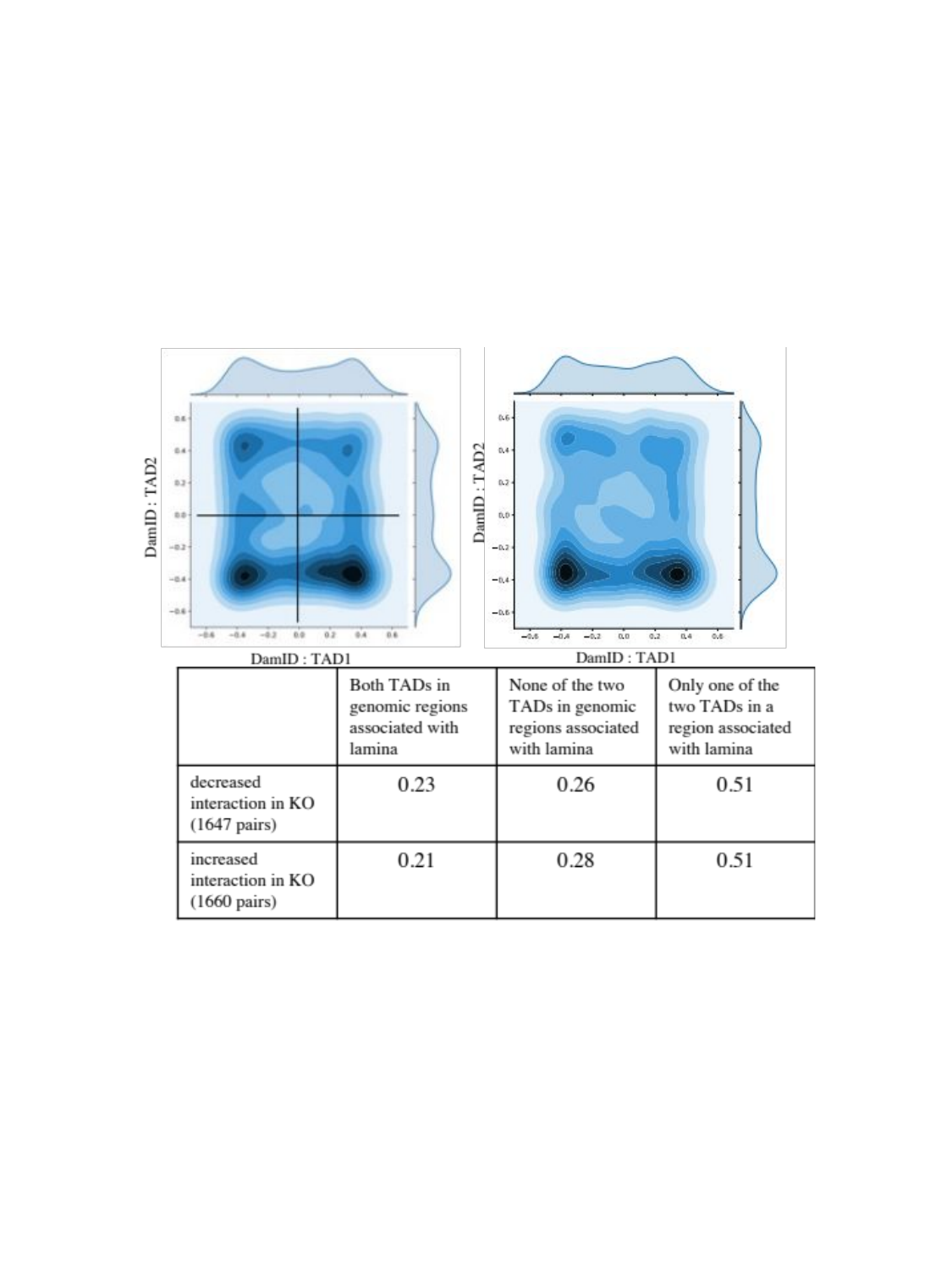

Supplement: Supplementary file 12 — Additional file 12: Figure S8. Simulated comparison of inter-TAD interactions. The analysis reported in panel C of Fig. 7 was repeated using a random control system where the boundary positions along the genome were permuted. [file 12915_2022_1375_MOESM12_ESM.pptx]
