# Supplementary material for: Hmga2 protein loss alters nuclear envelope and 3D chromatin structure
Source: BMC Biol. 2022 Aug 2;20:171. doi: 10.1186/s12915-022-01375-3 (PMC9344646; doi:10.1186/s12915-022-01375-3)
Supplement: Supplementary file 5 — Additional file 5: Figure S3. Lmnb1 abnormalities occur during the transition from the naïve state to the primed state of pluripotency. (A) Western blotting image showing Lmnb1 expression in Hmga2 wt and KO cells at day 3 after the induction of EpiLCs. Two different clones of PSCs were induced into EpiLCs for each condition. Western blotting quantification revealed no differences in terms of Lmnb1 expression between Hmga2 wild type and knock-out cells. The data in the graphs represent the mean ± SD of the Lmnb1 signal against Gapdh (n=3 biological replicates). ns: not significant (Student’s t-test, two tailed). (B) Lmnb1 immunofluorescence (red) on undifferentiated Hmga2 wt and KO cells showing no structural abnormalities of the nuclei. DAPI (blue) was used to counterstain the nuclei. A single plane of z-stack projection is shown. Scale bar = 50 μm. The data in the graphs represent the mean ± SD of the Lmnb1 signal against Gapdh (n=3 biological replicates). ns: not significant (Student’s t-test, two tailed). (C) q-PCR analysis to evaluate the levels of Hmga2 mRNA in EpiLCs upon transfection of siRNA. Non-silencing siRNA was used as control. The data in the graphs are reported as mean ± SD of relative values (fold changes) obtained normalizing the data of Hmga2 silencing with those of non-silencing control siRNA. **p<0.01, Student’s t-test, two tailed. (D) Western blotting image showing Hmga2 protein expression levels in EpiLCs upon transfection of non-silencing control siRNA and Hmga2 siRNA, respectively. [file 12915_2022_1375_MOESM5_ESM.pptx]

## Slide 1
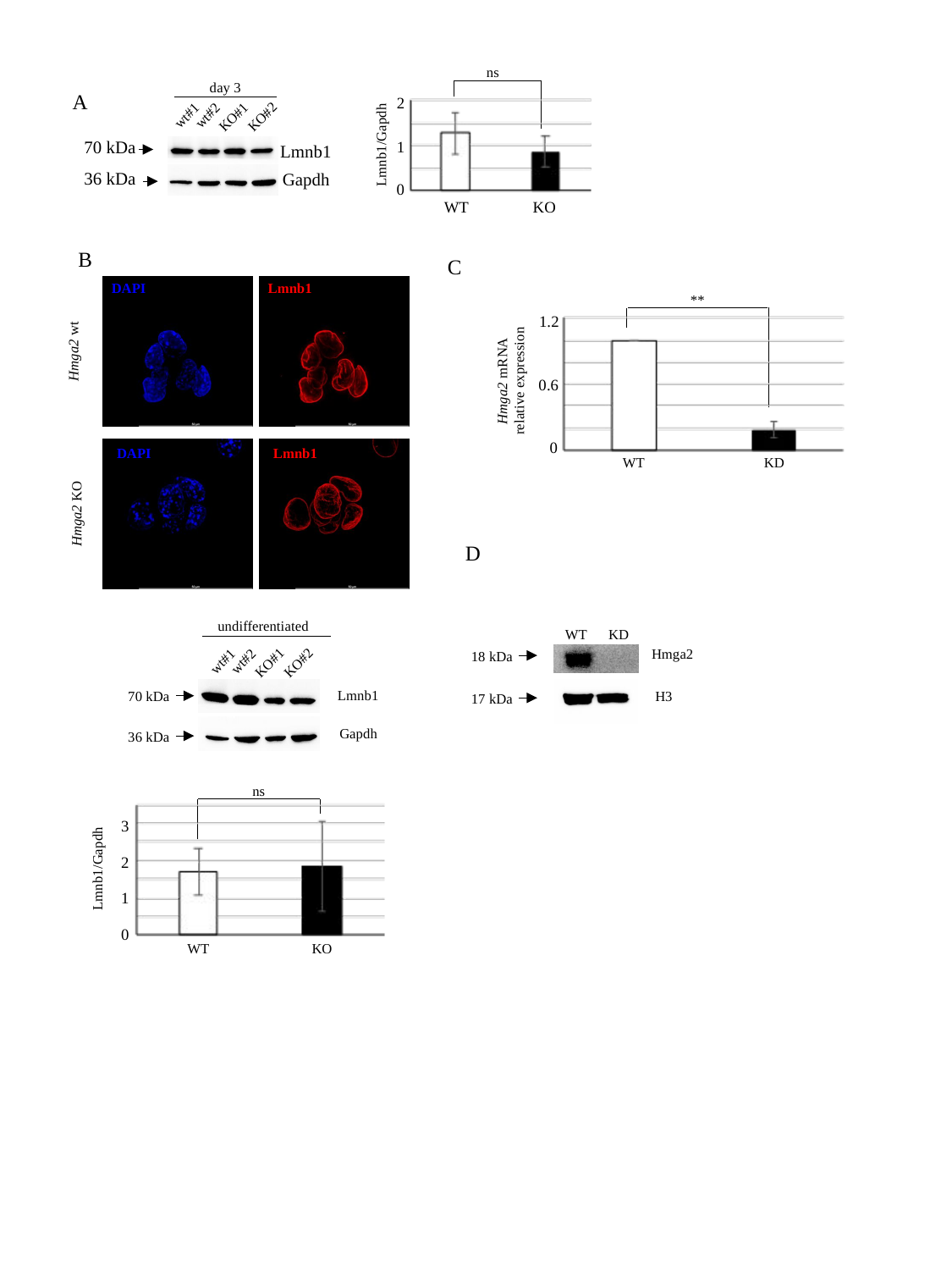

ns
day 3
A
2
wt#2
wt#1
KO#2
KO#1
70 kDa
Lmnb1/Gapdh
1
Lmnb1
36 kDa
Gapdh
0
WT KO
B
C
Lmnb1
DAPI
**
1.2
Hmga2 wt
 Hmga2 mRNA
 relative expression
0.6
0
Lmnb1
DAPI
WT
KD
Hmga2 KO
D
undifferentiated
Lmnb1
70 kDa
Gapdh
36 kDa
ns
Lmnb1/Gapdh
WT
KD
Hmga2
18 kDa
wt#2
wt#1
KO#2
KO#1
H3
17 kDa
3
2
1
0
WT
KO
