# Supplementary material for: Hmga2 protein loss alters nuclear envelope and 3D chromatin structure
Source: BMC Biol. 2022 Aug 2;20:171. doi: 10.1186/s12915-022-01375-3 (PMC9344646; doi:10.1186/s12915-022-01375-3)
Supplement: Supplementary file 7 — Additional file 7: Figure S5. Hmga2 wt and KO cells showed a similar localization of several histone H3 modifications upon the induction of EpiLCs. Immunoflurescence experiments on Hmga2 wt and KO cells at day 1 after the induction of EpiLCs. Lmnb1 (red) and H3K4me3, H3K9me2, H3K27me3 and H3K27ac (green) as indicated. Scale bars=50 μm. Quantification graphs obtained by counting >200 nuclei/condition. Error bars represent standard deviation. Statistical significance on three biological replicate experiments was determined using the student’s t-test, ns: not significant). [file 12915_2022_1375_MOESM7_ESM.pptx]

## Slide 1
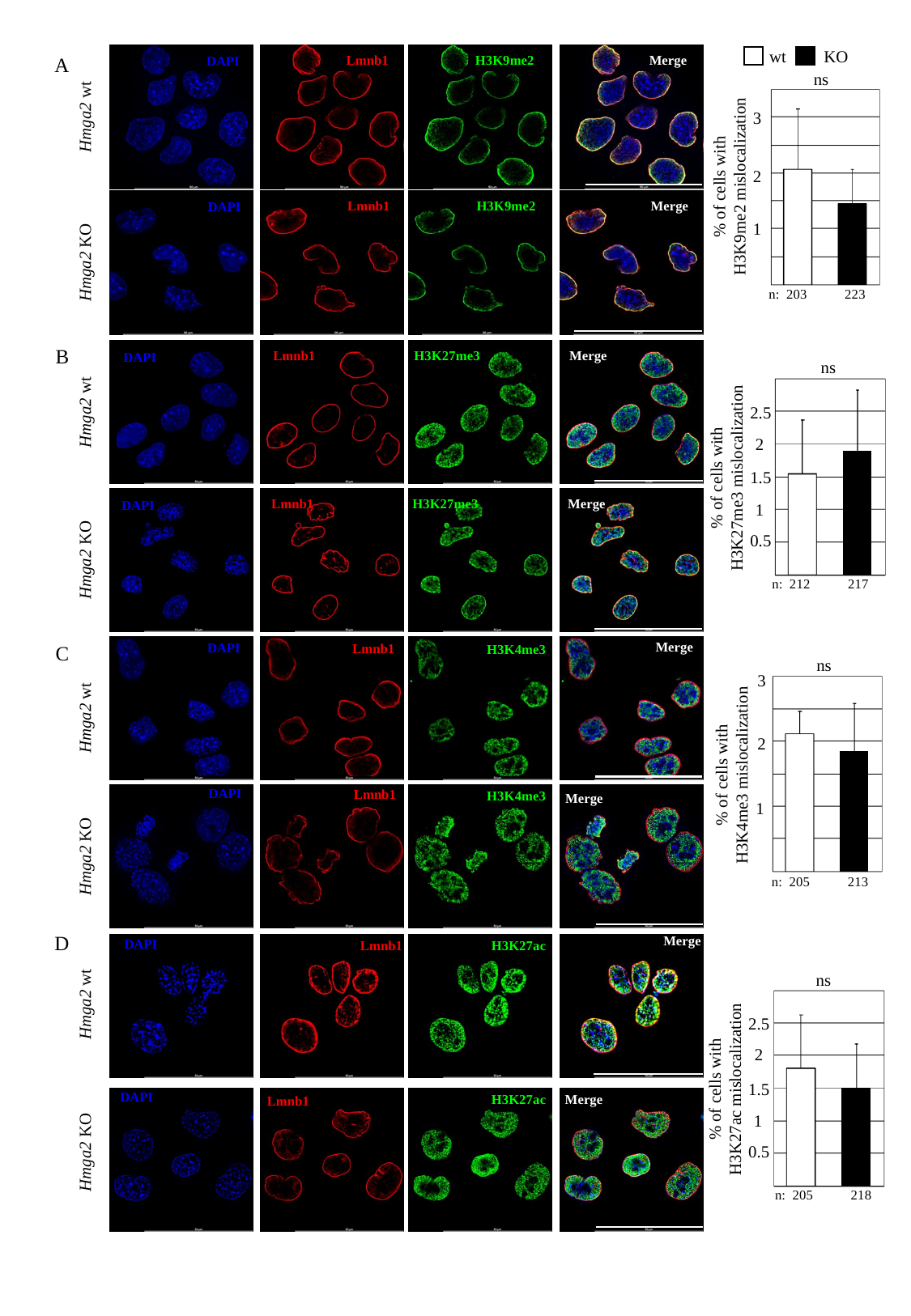

wt
KO
Lmnb1
H3K9me2
Merge
A
DAPI
ns
Hmga2 wt
3
2
% of cells with
H3K9me2 mislocalization
Lmnb1
H3K9me2
Merge
DAPI
1
Hmga2 KO
n: 203 223
B
Lmnb1
H3K27me3
Merge
DAPI
ns
2.5
Hmga2 wt
2
% of cells with
H3K27me3 mislocalization
1.5
Lmnb1
H3K27me3
Merge
DAPI
1
0.5
Hmga2 KO
n: 212 217
Merge
DAPI
Lmnb1
C
H3K4me3
ns
3
Hmga2 wt
2
% of cells with
H3K4me3 mislocalization
DAPI
Lmnb1
H3K4me3
Merge
1
Hmga2 KO
n: 205 213
D
Merge
DAPI
H3K27ac
Lmnb1
ns
Hmga2 wt
2.5
2
% of cells with
H3K27ac mislocalization
1.5
DAPI
H3K27ac
Merge
Lmnb1
1
0.5
Hmga2 KO
n: 205 218
