# Supplementary material for: Hmga2 protein loss alters nuclear envelope and 3D chromatin structure
Source: BMC Biol. 2022 Aug 2;20:171. doi: 10.1186/s12915-022-01375-3 (PMC9344646; doi:10.1186/s12915-022-01375-3)
Supplement: Supplementary file 8 — Additional file 8: Figure S6. Treatment of wt and KO cells with PRC2 inhibitor and its effect on nuclear lamina. (A) Western blotting image showing the level of H3K27me3 histone modifications in Hmga2 wt and KO PSCs treated or not with PRC2 inhibitor at day 0 and at day 1 after the induction of the transition into EpiLCs. The levels of the histone H3 were used as control. (B) Immunoflurescence experiments on Hmga2 wt and KO cells treated or not with PRC2 inhibitor at day 1 after the induction of EpiLCs showing the nuclear lamina phenotype. All pictures are shown as single z-plane, ROI 1024x1024, scale bar=50μm. Quantification graph showing the percentage of cells with nuclear lamina distortion in Hmga2 wt and KO cells treated or not with PRC2 inhibitor (n≤100 nuclei per condition). The count was performed at day 1 of EpiLC transition on three biological replicates. Error bars represent standard deviation. Statistical significance was determined using the student’s t-test (ns: not significant). [file 12915_2022_1375_MOESM8_ESM.pptx]

## Slide 1
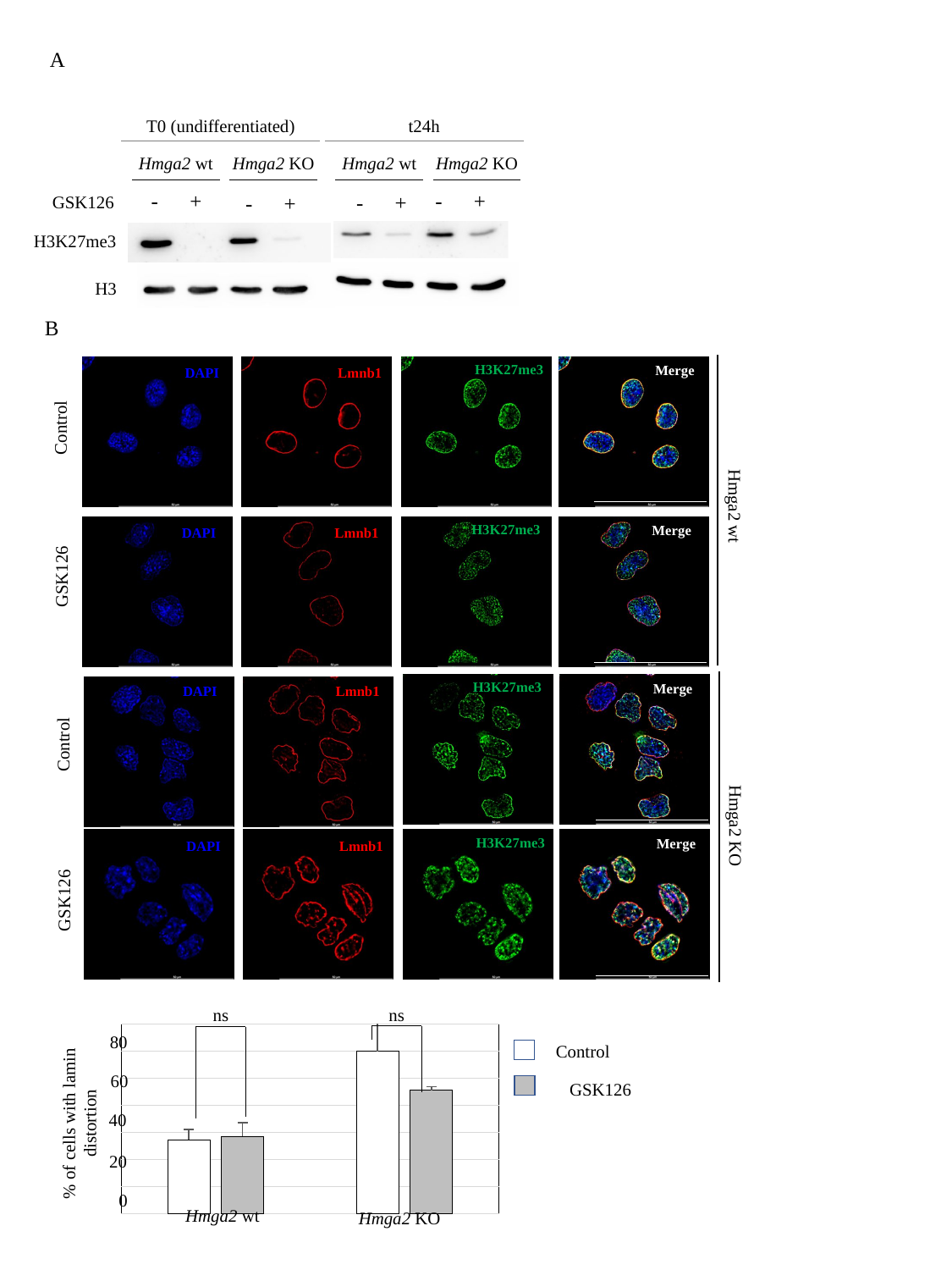

A
T0 (undifferentiated)
t24h
Hmga2 wt
Hmga2 KO
Hmga2 wt
Hmga2 KO
- +
- +
- +
- +
GSK126
H3K27me3
H3
B
H3K27me3
Merge
DAPI
Lmnb1
Control
Hmga2 wt
H3K27me3
Merge
DAPI
Lmnb1
GSK126
H3K27me3
Merge
DAPI
Lmnb1
Control
Hmga2 KO
H3K27me3
Merge
DAPI
Lmnb1
GSK126
ns
ns
### Chart
| Category | Untreated | PRC2 inhibitor |
|---|---|---|80
Control
60
GSK126
% of cells with lamin distortion
40
20
0
Hmga2 wt
Hmga2 KO
