# Supplementary material for: Hmga2 protein loss alters nuclear envelope and 3D chromatin structure
Source: BMC Biol. 2022 Aug 2;20:171. doi: 10.1186/s12915-022-01375-3 (PMC9344646; doi:10.1186/s12915-022-01375-3)
Supplement: Supplementary file 10 — Additional file 10: Figure S7. Identification of A/B compartment. (A) 1st eigenvector from the PCA analysis is shown in wt and KO cells. GC content (reported below) is used to call A/B compartment. All signals are binned at 100kb. (B) Pearson correlation between the 1st eigenvector in wt and KO for each chromosome. Dashed line represents the average value. (C) Examples of genomic regions exhibiting changes in A/B compartment membership from wt to KO condition. [file 12915_2022_1375_MOESM10_ESM.pptx]

## Slide 1
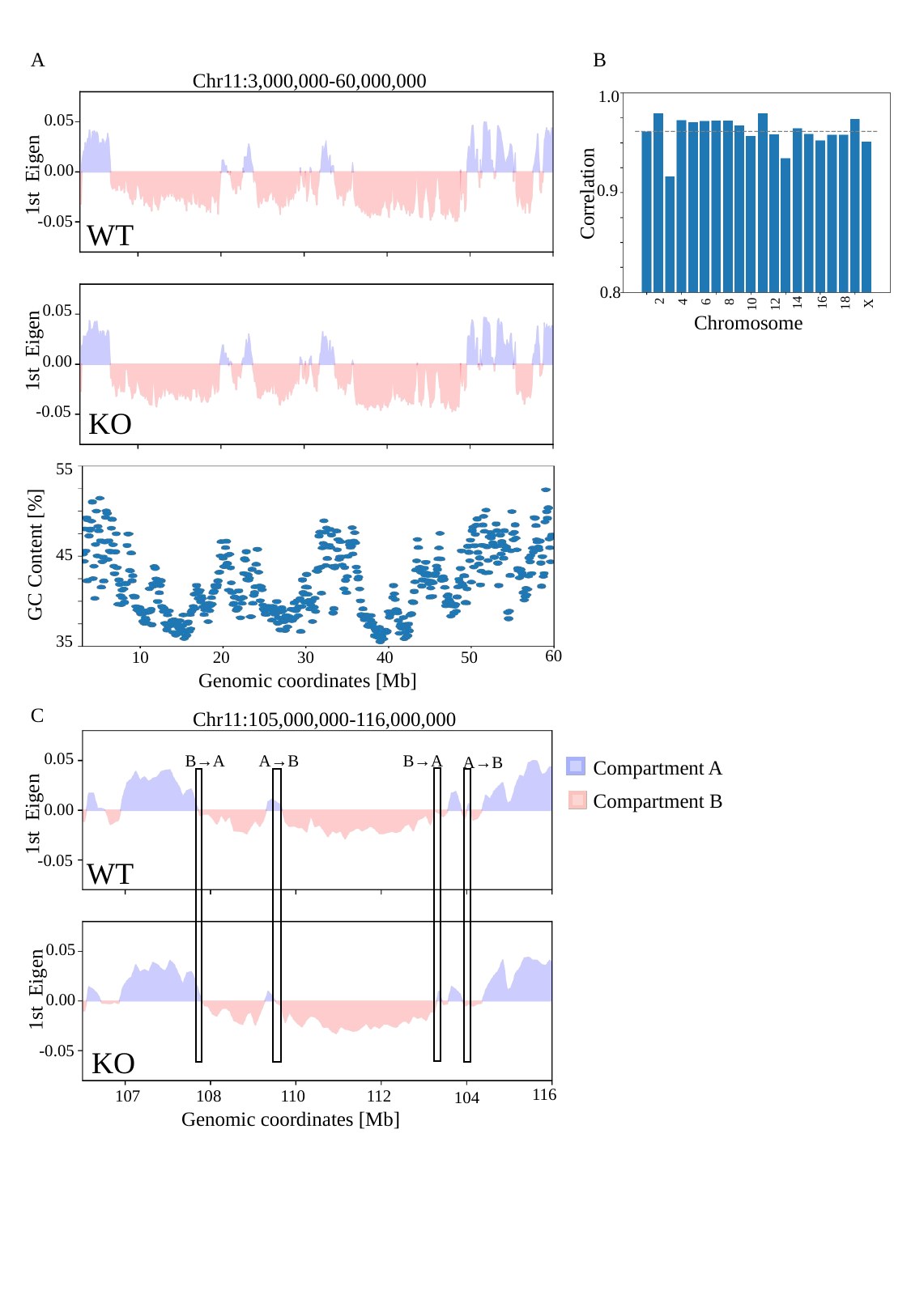

A
B
Chr11:3,000,000-60,000,000
1.0
0.05
0.00
1st Eigen
0.9
Correlation
-0.05
WT
0.8
2
4
6
8
14
16
18
X
12
10
0.05
Chromosome
1st Eigen
0.00
-0.05
KO
55
GC Content [%]
45
35
60
10
20
30
40
50
Genomic coordinates [Mb]
C
Chr11:105,000,000-116,000,000
0.05
A→B
B→A
B→A
A→B
Compartment A
Compartment B
0.00
1st Eigen
-0.05
WT
0.05
1st Eigen
0.00
-0.05
KO
116
107
108
110
112
104
Genomic coordinates [Mb]
