# Supplementary material for: Hmga2 protein loss alters nuclear envelope and 3D chromatin structure
Source: BMC Biol. 2022 Aug 2;20:171. doi: 10.1186/s12915-022-01375-3 (PMC9344646; doi:10.1186/s12915-022-01375-3)
Supplement: Supplementary file 13 — Additional file 13. Uncropped blots. [file 12915_2022_1375_MOESM13_ESM.pptx]

## Slide 1
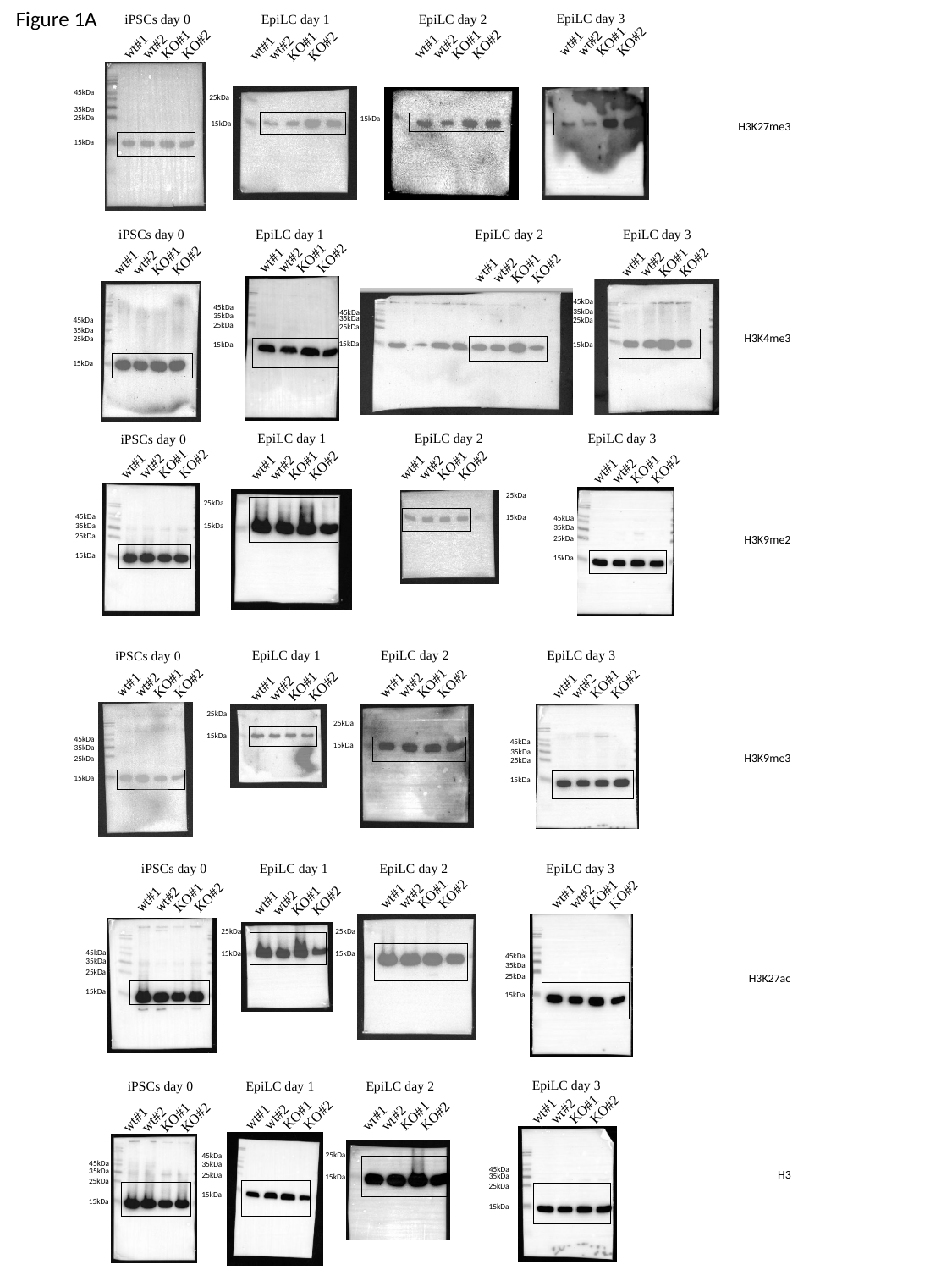

EpiLC day 3
EpiLC day 1
EpiLC day 2
iPSCs day 0
Figure 1A
KO#1
KO#2
wt#1
wt#2
KO#1
KO#2
wt#1
wt#2
KO#1
KO#2
wt#1
wt#2
KO#1
KO#2
wt#1
wt#2
45kDa
25kDa
35kDa
25kDa
H3K27me3
15kDa
15kDa
15kDa
EpiLC day 3
EpiLC day 1
EpiLC day 2
iPSCs day 0
KO#1
KO#2
wt#1
wt#2
KO#1
KO#2
wt#1
wt#2
KO#1
KO#2
wt#1
wt#2
KO#1
KO#2
wt#1
wt#2
45kDa
45kDa
35kDa
45kDa
35kDa
35kDa
45kDa
25kDa
25kDa
25kDa
35kDa
H3K4me3
25kDa
15kDa
15kDa
15kDa
15kDa
EpiLC day 3
EpiLC day 1
EpiLC day 2
iPSCs day 0
KO#1
KO#2
wt#1
wt#2
KO#1
KO#2
wt#1
wt#2
KO#1
KO#2
wt#1
wt#2
KO#1
KO#2
wt#1
wt#2
25kDa
25kDa
45kDa
15kDa
45kDa
35kDa
15kDa
35kDa
H3K9me2
25kDa
25kDa
15kDa
15kDa
EpiLC day 3
EpiLC day 1
EpiLC day 2
iPSCs day 0
KO#1
KO#2
wt#1
wt#2
KO#1
KO#2
wt#1
wt#2
KO#1
KO#2
wt#1
wt#2
KO#1
KO#2
wt#1
wt#2
25kDa
25kDa
15kDa
45kDa
45kDa
15kDa
35kDa
H3K9me3
35kDa
25kDa
25kDa
15kDa
15kDa
EpiLC day 3
EpiLC day 1
EpiLC day 2
iPSCs day 0
KO#1
KO#2
wt#1
wt#2
KO#1
KO#2
wt#1
wt#2
KO#1
KO#2
wt#1
wt#2
KO#1
KO#2
wt#1
wt#2
25kDa
25kDa
45kDa
15kDa
15kDa
45kDa
35kDa
35kDa
H3K27ac
25kDa
25kDa
15kDa
15kDa
EpiLC day 3
EpiLC day 1
EpiLC day 2
iPSCs day 0
KO#1
KO#2
wt#1
wt#2
KO#1
KO#2
wt#1
wt#2
KO#1
KO#2
wt#1
wt#2
KO#1
KO#2
wt#1
wt#2
25kDa
45kDa
45kDa
35kDa
H3
45kDa
35kDa
25kDa
35kDa
15kDa
25kDa
25kDa
15kDa
15kDa
15kDa

## Slide 2
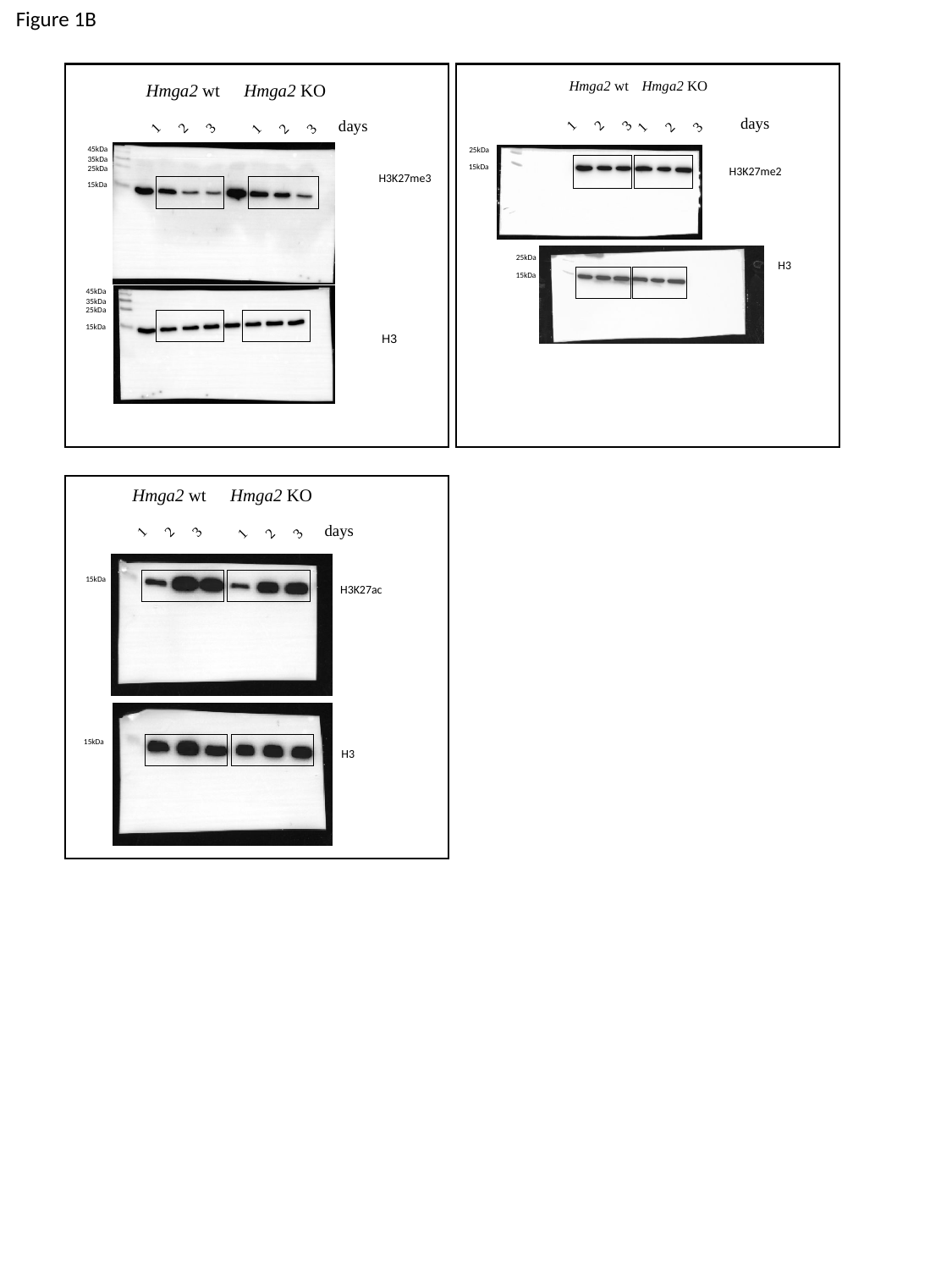

Figure 1B
Hmga2 wt
Hmga2 KO
Hmga2 wt
Hmga2 KO
days
days
2
1
3
2
3
1
2
1
3
2
3
1
45kDa
25kDa
35kDa
H3K27me2
15kDa
25kDa
H3K27me3
15kDa
25kDa
H3
15kDa
45kDa
35kDa
25kDa
15kDa
H3
Hmga2 wt
Hmga2 KO
days
2
1
3
2
3
1
15kDa
H3K27ac
15kDa
H3

## Slide 3
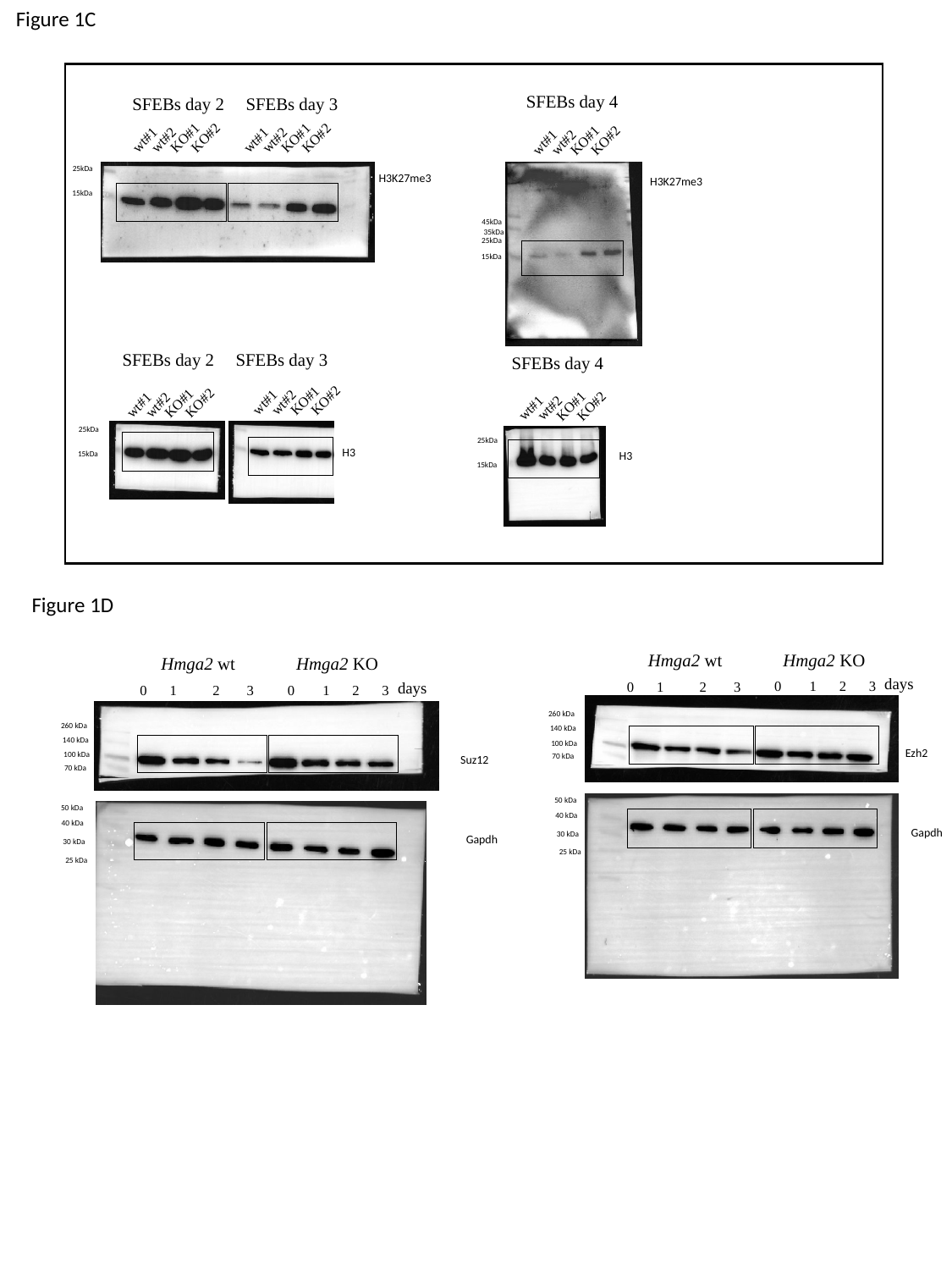

Figure 1C
SFEBs day 4
SFEBs day 2
SFEBs day 3
KO#1
KO#2
wt#1
wt#2
KO#1
KO#2
wt#1
wt#2
KO#1
KO#2
wt#1
wt#2
25kDa
H3K27me3
H3K27me3
15kDa
45kDa
35kDa
25kDa
15kDa
SFEBs day 2
SFEBs day 3
SFEBs day 4
KO#1
KO#2
wt#1
wt#2
KO#1
KO#2
wt#1
wt#2
KO#1
KO#2
wt#1
wt#2
25kDa
25kDa
H3
H3
15kDa
15kDa
Figure 1D
Hmga2 wt
Hmga2 KO
Hmga2 wt
Hmga2 KO
days
0
1
2
3
days
0
1
2
3
0
1
2
3
0
1
2
3
260 kDa
260 kDa
140 kDa
140 kDa
100 kDa
Ezh2
Suz12
100 kDa
70 kDa
70 kDa
50 kDa
50 kDa
40 kDa
40 kDa
Gapdh
Gapdh
30 kDa
30 kDa
25 kDa
25 kDa

## Slide 4
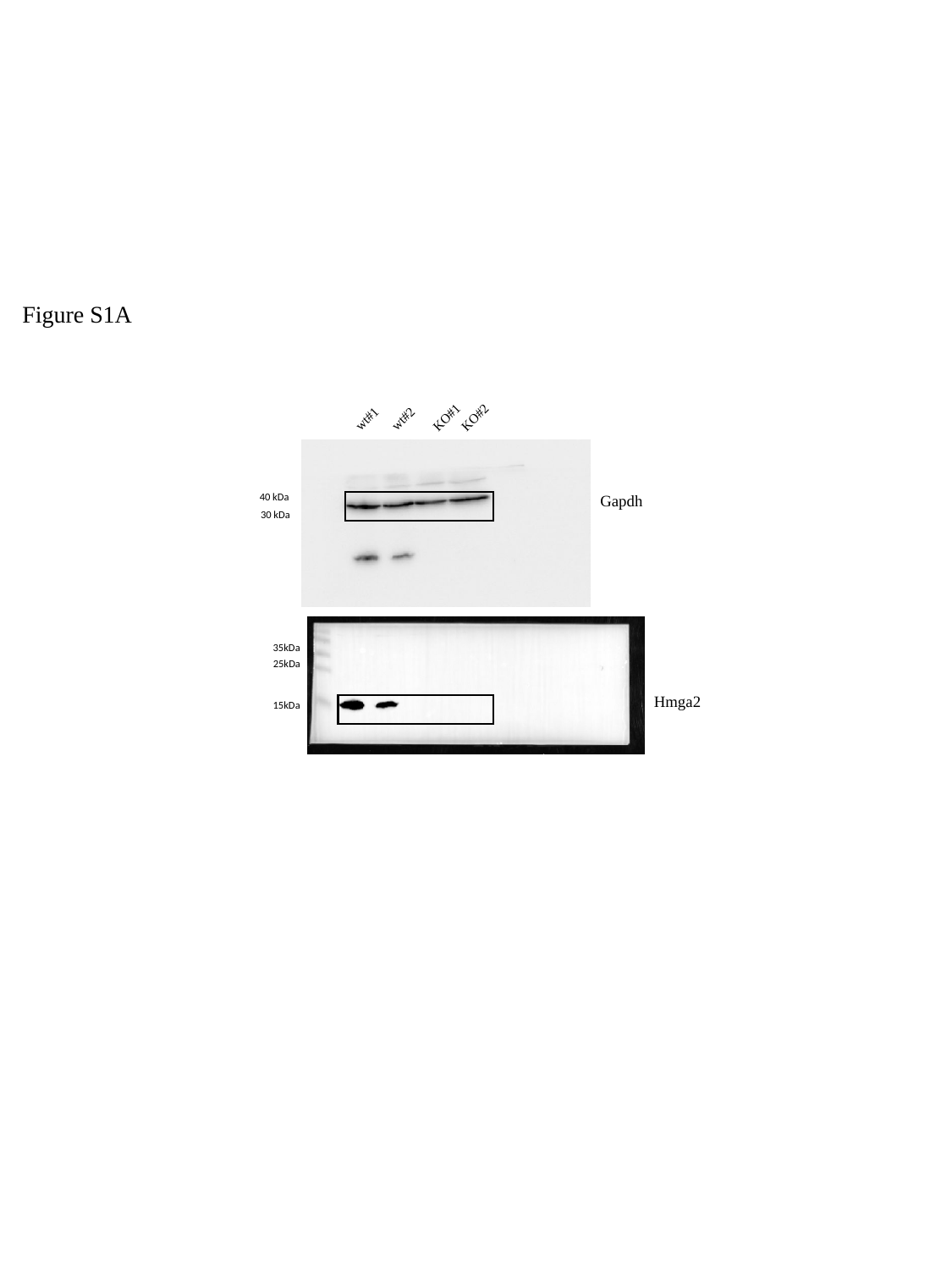

Figure S1A
KO#1
KO#2
wt#1
wt#2
40 kDa
Gapdh
30 kDa
35kDa
25kDa
15kDa
Hmga2

## Slide 5
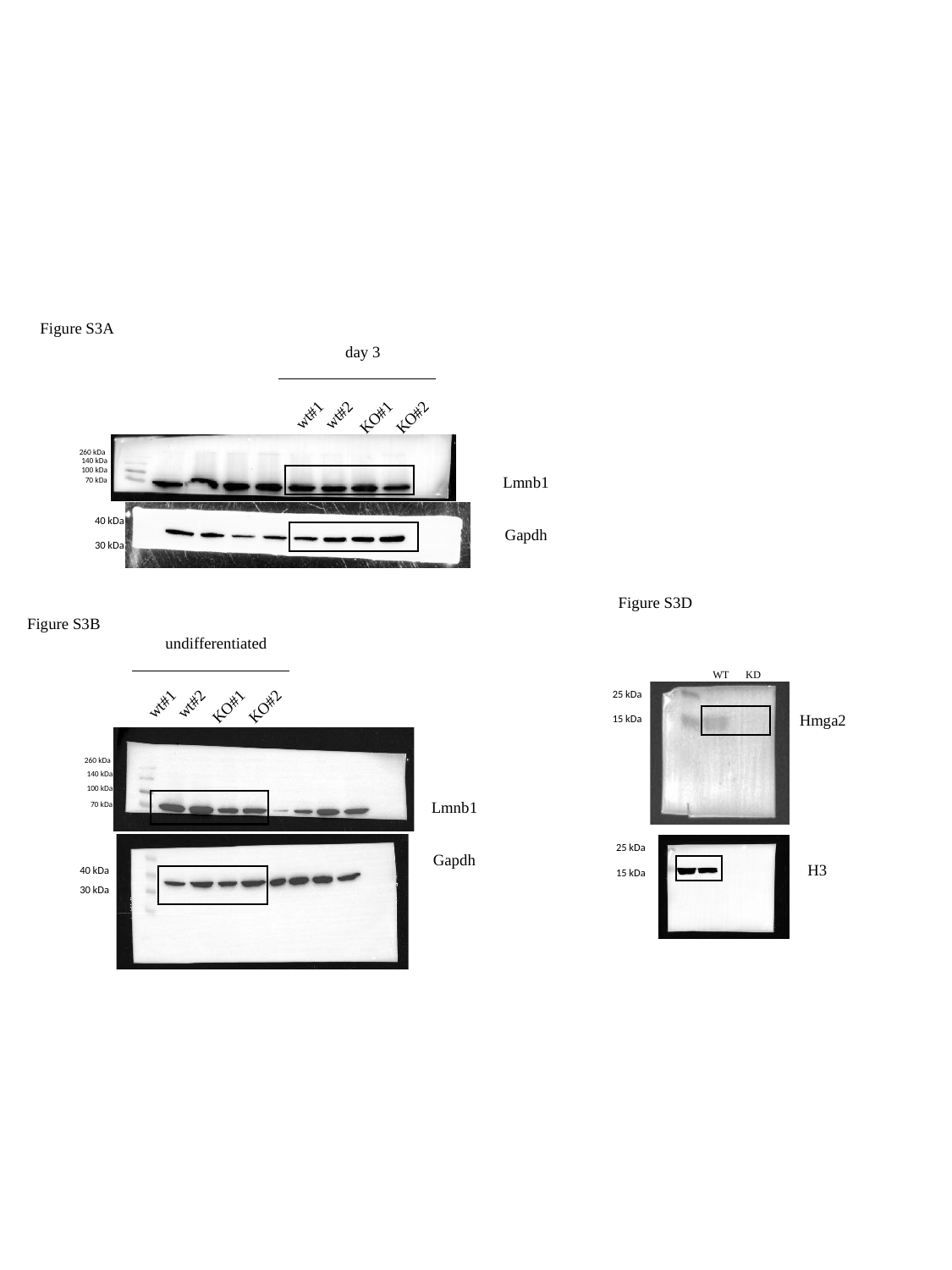

Figure S3A
day 3
wt#2
wt#1
KO#1
KO#2
260 kDa
140 kDa
100 kDa
70 kDa
Lmnb1
40 kDa
Gapdh
30 kDa
Figure S3D
Figure S3B
undifferentiated
WT
KD
25 kDa
wt#2
wt#1
KO#1
KO#2
15 kDa
Hmga2
260 kDa
140 kDa
100 kDa
70 kDa
Lmnb1
25 kDa
Gapdh
40 kDa
15 kDa
H3
30 kDa

## Slide 6
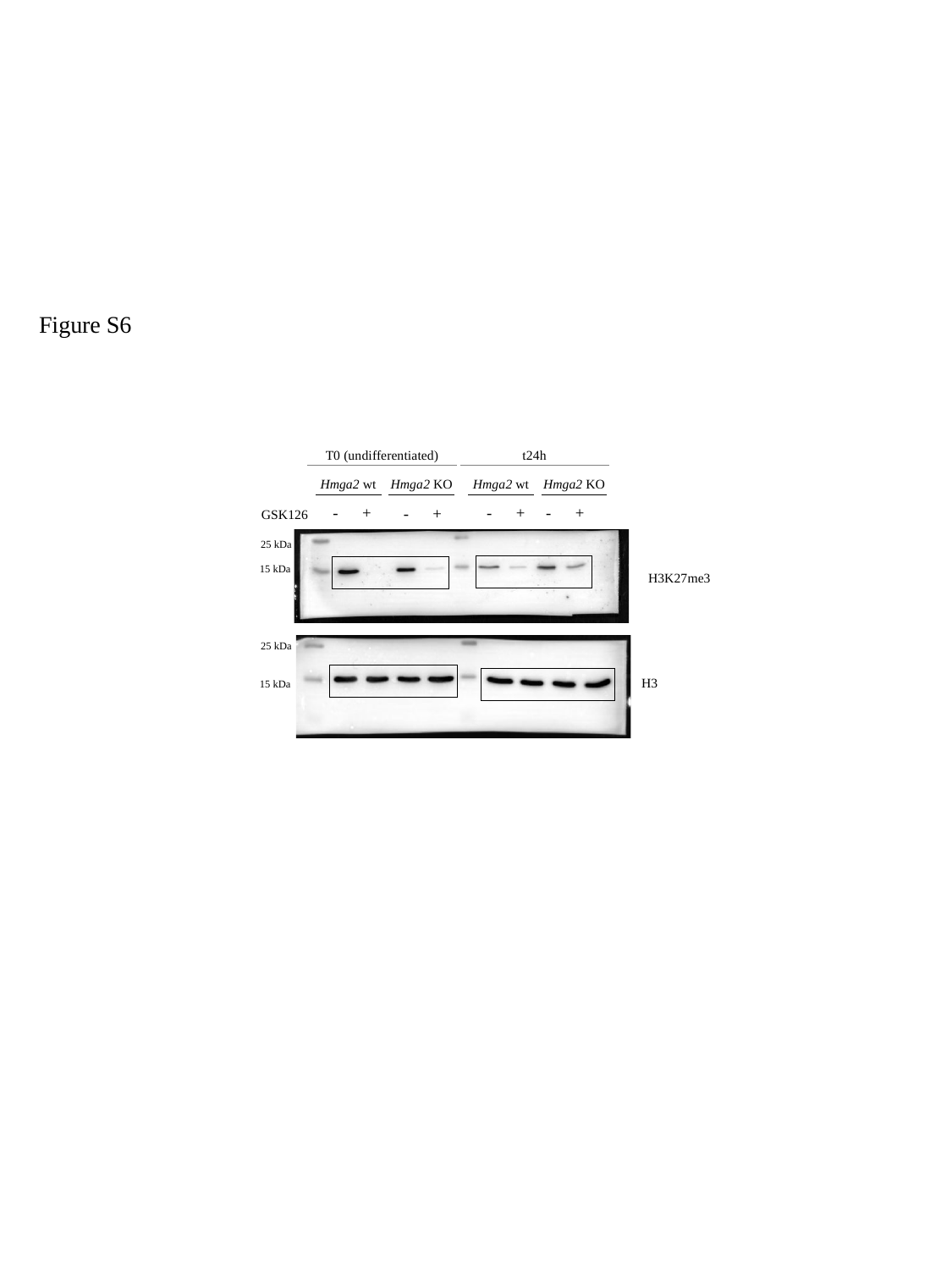

Figure S6
T0 (undifferentiated)
t24h
Hmga2 wt
Hmga2 KO
Hmga2 wt
Hmga2 KO
- +
- +
- +
- +
GSK126
25 kDa
15 kDa
H3K27me3
25 kDa
H3
15 kDa
